# Supplementary figures and images for: Comprehensive Longitudinal Microbiome Analysis of the Chicken Cecum Reveals a Shift From Competitive to Environmental Drivers and a Window of Opportunity for Campylobacter
Source: Front Microbiol. 2018 Oct 15;9:2452. doi: 10.3389/fmicb.2018.02452 (PMC6196313; doi:10.3389/fmicb.2018.02452)

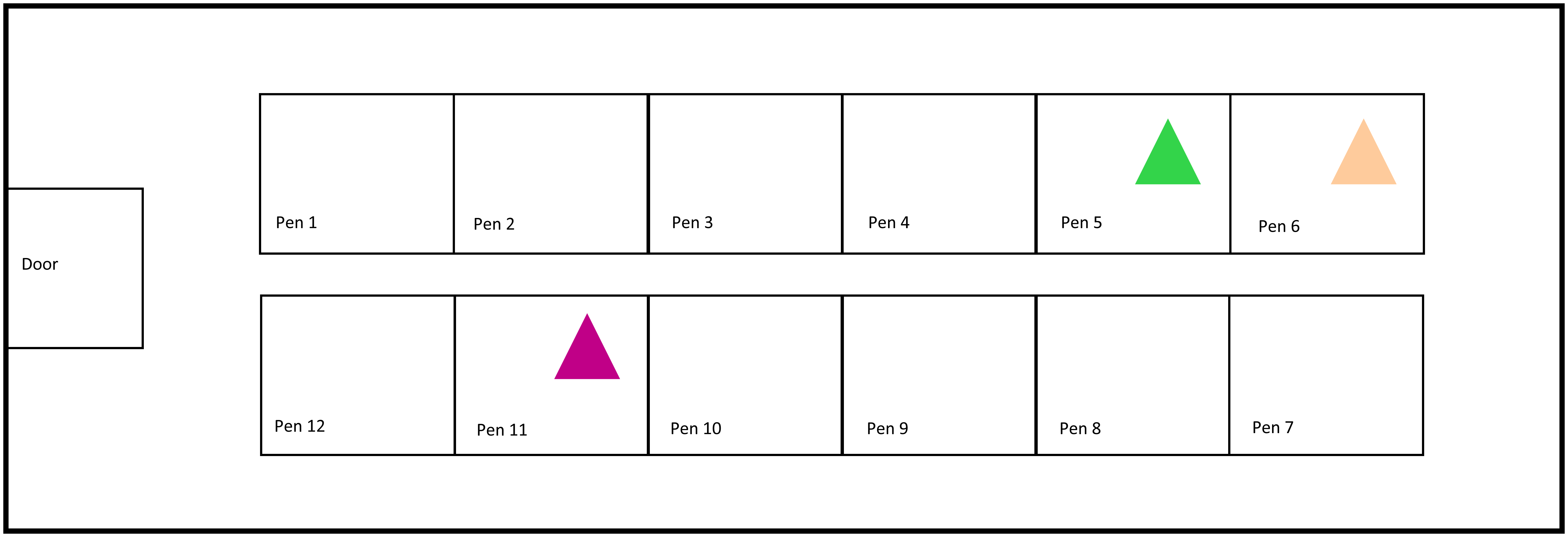

Supplement: Supplementary Figure 1 — Spatial arrangement of pens. Triangles indicate pens with Campylobacter. [file Image_1.TIFF]
