# Supplementary material for: Comprehensive Longitudinal Microbiome Analysis of the Chicken Cecum Reveals a Shift From Competitive to Environmental Drivers and a Window of Opportunity for Campylobacter
Source: Front Microbiol. 2018 Oct 15;9:2452. doi: 10.3389/fmicb.2018.02452 (PMC6196313; doi:10.3389/fmicb.2018.02452)
Supplement: Supplementary Table 1 — Differential analysis of OTUs that are up/down-regulated between different groups (Adjusted P values ≤ 0.05) where positive log2 fold change represent OTUs becoming abundant as we go forward in time. Here only the significant OTUs are shown for both daily and weekly comparisons. [file Table_1.DOCX]

Supplementary Table 1: Differential analysis of OTUs that are up/down-regulated between different groups (Adjusted P values ≤ 0.05) where positive log2 fold change represent OTUs becoming abundant as we go forward in time. Here only the significant OTUs are shown for both daily and weekly comparisons.

| OTUs | Base Mean Abundance | Log2 Fold Change | Group Comparison |
| --- | --- | --- | --- |
| OTU_46 Bacteria;Firmicutes;Clostridia;Clostridiales;Ruminococcaceae;Anaerotruncus | 85.84 | 2.11 | 03-04 |
| OTU_504 No blast hit | 10.63 | -2.01 | 03-04 |
| OTU_50 Bacteria;Firmicutes;Clostridia;Clostridiales;Ruminococcaceae;Ruminococcaceae UCG-014 | 20.53 | -2.24 | 04-05 |
| OTU_1186 Bacteria;Firmicutes;Clostridia;Clostridiales;Lachnospiraceae;Roseburia | 9.07 | 2.40 | 09-10 |
| OTU_1560 Bacteria;Firmicutes;Clostridia;Clostridiales;Lachnospiraceae;Lachnoclostridium | 1405.48 | 2.11 | 09-10 |
| OTU_859 No blast hit | 5.11 | 2.22 | 09-10 |
| OTU_1657 Bacteria;Firmicutes;Clostridia;Clostridiales;Lachnospiraceae;Fusicatenibacter | 4.98 | 2.12 | 09-10 |
| OTU_186 Bacteria;Firmicutes;Clostridia;Clostridiales;Ruminococcaceae;Ruminococcaceae UCG-005 | 28.35 | 2.19 | 09-10 |
| OTU_1092 No blast hit | 4.39 | 2.05 | 09-10 |
| OTU_4034 Bacteria;Firmicutes;Clostridia;Clostridiales;Lachnospiraceae;Fusicatenibacter | 5.71 | 2.10 | 09-10 |
| OTU_537 Bacteria;Firmicutes;Clostridia;Clostridiales;Ruminococcaceae;Ruminiclostridium 5 | 4.95 | 2.05 | 10-11 |
| OTU_196 Bacteria;Firmicutes;Clostridia;Clostridiales;Ruminococcaceae | 21.31 | 2.29 | 10-11 |
| OTU_3453 Bacteria;Firmicutes;Clostridia;Clostridiales;Lachnospiraceae;Tyzzerella | 145.35 | 2.32 | 10-11 |
| OTU_126 Bacteria;Firmicutes;Clostridia;Clostridiales;Ruminococcaceae;Ruminococcaceae NK4A214 group | 7.97 | 2.34 | 10-11 |
| OTU_178 Bacteria;Firmicutes;Clostridia;Clostridiales;Ruminococcaceae;Anaerotruncus | 19.30 | 3.14 | 10-11 |
| OTU_13945 Bacteria;Firmicutes;Clostridia;Clostridiales;Ruminococcaceae;Ruminiclostridium 9 | 18.09 | -2.92 | 10-11 |
| OTU_11096 Bacteria;Firmicutes;Clostridia;Clostridiales;Ruminococcaceae | 7.83 | -2.30 | 10-11 |
| OTU_16519 Bacteria;Firmicutes;Clostridia;Clostridiales;Ruminococcaceae | 7.75 | -2.29 | 10-11 |
| OTU_155 Bacteria;Firmicutes;Clostridia;Clostridiales;Ruminococcaceae;Ruminococcaceae UCG-014 | 27.48 | -2.25 | 10-11 |
| OTU_99 Bacteria;Firmicutes;Clostridia;Clostridiales;Clostridiales vadinBB60 group | 46.84 | -2.24 | 10-11 |
| OTU_4425 Bacteria;Firmicutes;Clostridia;Clostridiales;Clostridiales vadinBB60 group | 53.73 | -2.24 | 10-11 |
| OTU_2264 Bacteria;Firmicutes;Clostridia;Clostridiales;Ruminococcaceae;Ruminiclostridium 9 | 12.43 | -2.23 | 10-11 |
| OTU_4403 Bacteria;Firmicutes;Clostridia;Clostridiales;Lachnospiraceae;Lachnoclostridium | 7.48 | -2.21 | 10-11 |
| OTU_273 Bacteria;Tenericutes;Mollicutes;Mollicutes RF9 | 9.76 | -2.20 | 10-11 |
| OTU_81 Bacteria;Firmicutes;Clostridia;Clostridiales;Lachnospiraceae;Fusicatenibacter | 650.85 | -2.17 | 10-11 |
| OTU_17303 Bacteria;Firmicutes;Clostridia;Clostridiales;Clostridiales vadinBB60 group | 23.24 | -2.17 | 10-11 |
| OTU_38 Bacteria;Firmicutes;Clostridia;Clostridiales;Ruminococcaceae | 678.29 | -2.14 | 10-11 |
| OTU_88 Bacteria;Firmicutes;Clostridia;Clostridiales;Lachnospiraceae;Lachnospiraceae FE2018 group | 496.93 | -2.11 | 10-11 |
| OTU_26 Bacteria;Firmicutes;Clostridia;Clostridiales;Lachnospiraceae;Lachnoclostridium | 5197.77 | -2.06 | 10-11 |
| OTU_1657 Bacteria;Firmicutes;Clostridia;Clostridiales;Lachnospiraceae;Fusicatenibacter | 6.10 | -2.06 | 10-11 |
| OTU_4034 Bacteria;Firmicutes;Clostridia;Clostridiales;Lachnospiraceae;Fusicatenibacter | 7.03 | -2.04 | 10-11 |
| OTU_8773 Bacteria;Firmicutes;Clostridia;Clostridiales;Ruminococcaceae;[Eubacterium] coprostanoligenes group | 27.71 | 2.03 | 12-13 |
| OTU_669 No blast hit | 8.63 | -2.03 | 13-14 |
| OTU_5854 Bacteria;Firmicutes;Bacilli;Lactobacillales;Lactobacillaceae;Lactobacillus | 277.21 | 2.44 | 14-15 |
| OTU_56 Bacteria;Firmicutes;Bacilli;Lactobacillales;Lactobacillaceae;Lactobacillus | 446.98 | 2.41 | 14-15 |
| OTU_157 Bacteria;Firmicutes;Bacilli;Lactobacillales;Lactobacillaceae;Lactobacillus | 225.97 | 2.43 | 14-15 |
| OTU_302 Bacteria;Firmicutes;Clostridia;Clostridiales;Lachnospiraceae;Lachnospiraceae FCS020 group | 86.06 | 2.37 | 14-15 |
| OTU_152 Bacteria;Firmicutes;Clostridia;Clostridiales;Ruminococcaceae;Ruminococcaceae UCG-014 | 29.02 | 2.38 | 14-15 |
| OTU_149 Bacteria;Firmicutes;Clostridia;Clostridiales;Lachnospiraceae;Lachnoclostridium | 182.29 | 2.34 | 14-15 |
| OTU_258 No blast hit | 11.99 | 2.28 | 14-15 |
| OTU_3293 Bacteria;Firmicutes;Clostridia;Clostridiales;Ruminococcaceae;Ruminiclostridium 5 | 318.03 | 2.20 | 14-15 |
| OTU_33 Bacteria;Firmicutes;Clostridia;Clostridiales;Ruminococcaceae;Ruminiclostridium 5 | 815.02 | 2.14 | 14-15 |
| OTU_357 Bacteria;Firmicutes;Clostridia;Clostridiales;Lachnospiraceae;Blautia | 21.50 | 2.10 | 14-15 |
| OTU_86 Bacteria;Firmicutes;Bacilli;Lactobacillales;Streptococcaceae;Streptococcus | 11.32 | 2.19 | 15-16 |
| OTU_140 Bacteria;Firmicutes;Clostridia;Clostridiales;Ruminococcaceae;Ruminococcaceae UCG-004 | 12.64 | 2.17 | 15-16 |
| OTU_94 Bacteria;Firmicutes;Clostridia;Clostridiales;Ruminococcaceae;Ruminococcaceae UCG-014 | 277.06 | 2.06 | 15-16 |
| OTU_302 Bacteria;Firmicutes;Clostridia;Clostridiales;Lachnospiraceae;Lachnospiraceae FCS020 group | 62.39 | -2.00 | 16-17 |
| OTU_204 Bacteria;Firmicutes;Clostridia;Clostridiales;Lachnospiraceae;Tyzzerella 3;[Clostridium] colinum | 29.20 | 2.49 | 17-18 |
| OTU_602 Bacteria;Firmicutes;Clostridia;Clostridiales;Ruminococcaceae;Ruminococcaceae UCG-014 | 94.56 | 2.40 | 18-19 |
| OTU_13399 Bacteria;Firmicutes;Clostridia;Clostridiales;Ruminococcaceae;Ruminococcaceae UCG-002 | 10.76 | 2.07 | 18-19 |
| OTU_66 Bacteria;Firmicutes;Clostridia;Clostridiales;Ruminococcaceae | 25.13 | 2.02 | 18-19 |
| OTU_201 Bacteria;Firmicutes;Clostridia;Clostridiales;Clostridiales vadinBB60 group | 17.76 | 2.11 | 19-20 |
| OTU_459 Bacteria;Firmicutes;Clostridia;Clostridiales;Ruminococcaceae;Ruminococcaceae UCG-004 | 7.79 | 2.16 | 19-20 |
| OTU_929 Bacteria;Firmicutes;Clostridia;Clostridiales;Ruminococcaceae;Ruminococcaceae UCG-004 | 10.94 | 2.40 | 19-20 |
| OTU_630 Bacteria;Firmicutes;Clostridia;Clostridiales;Ruminococcaceae;Intestinimonas | 22.84 | 2.42 | 19-20 |
| OTU_4 Bacteria;Proteobacteria;Gammaproteobacteria;Enterobacteriales;Enterobacteriaceae;Escherichia-Shigella | 1987.33 | -2.25 | 19-20 |
| OTU_5864 Bacteria;Firmicutes;Clostridia;Clostridiales;Ruminococcaceae;Ruminococcaceae UCG-014 | 302.73 | -2.21 | 19-20 |
| OTU_229 Bacteria;Firmicutes;Clostridia;Clostridiales;Clostridiales vadinBB60 group | 13.95 | 2.13 | 20-21 |
| OTU_287 Bacteria;Firmicutes;Clostridia;Clostridiales;Ruminococcaceae;Ruminococcaceae UCG-014 | 65.90 | -2.13 | 20-21 |
| OTU_118 Bacteria;Firmicutes;Clostridia;Clostridiales;Clostridiales vadinBB60 group | 195.16 | -2.07 | 20-21 |
| OTU_272 Bacteria;Firmicutes;Clostridia;Clostridiales;Ruminococcaceae;Ruminococcaceae UCG-013 | 26.90 | 2.31 | 21-22 |
| OTU_2921 Bacteria;Firmicutes;Clostridia;Clostridiales;Ruminococcaceae;Ruminococcaceae UCG-005 | 6.80 | -2.17 | 21-22 |
| OTU_743 Bacteria;Firmicutes;Clostridia;Clostridiales;Ruminococcaceae | 5.46 | -2.03 | 21-22 |
| OTU_476 No blast hit | 8.71 | -2.01 | 21-22 |
| OTU_293 Bacteria;Firmicutes;Clostridia;Clostridiales;Clostridiales vadinBB60 group | 24.80 | -2.17 | 22-23 |
| OTU_71 Bacteria;Actinobacteria;Coriobacteriia;Coriobacteriales;Coriobacteriaceae;Olsenella | 28.83 | 2.26 | 23-24 |
| OTU_3471 Bacteria;Actinobacteria;Coriobacteriia;Coriobacteriales;Coriobacteriaceae;Olsenella | 17.11 | 2.30 | 23-24 |
| OTU_165 Bacteria;Firmicutes;Clostridia;Clostridiales;Ruminococcaceae;Ruminococcaceae UCG-014 | 18.76 | 2.16 | 23-24 |
| OTU_6645 Bacteria;Firmicutes;Clostridia;Clostridiales;Clostridiales vadinBB60 group | 16.81 | 2.14 | 23-24 |
| OTU_240 Bacteria;Firmicutes;Clostridia;Clostridiales;Ruminococcaceae;Ruminococcaceae UCG-014 | 66.78 | 2.13 | 23-24 |
| OTU_13523 Bacteria;Firmicutes;Bacilli;Bacillales;Bacillaceae;Bacillus | 137.89 | 2.01 | 23-24 |
| OTU_37 Bacteria;Firmicutes;Clostridia;Clostridiales;Clostridiales vadinBB60 group | 310.22 | 2.12 | 23-24 |
| OTU_341 Bacteria;Firmicutes;Clostridia;Clostridiales;Ruminococcaceae;Ruminococcaceae UCG-014 | 12.91 | 2.25 | 24-25 |
| OTU_288 Bacteria;Firmicutes;Clostridia;Clostridiales;Ruminococcaceae;Anaerotruncus | 29.58 | 2.07 | 25-26 |
| OTU_552 Bacteria;Firmicutes;Clostridia;Clostridiales;Ruminococcaceae | 326.78 | 2.10 | 25-26 |
| OTU_2701 Bacteria;Firmicutes;Clostridia;Clostridiales;Lachnospiraceae;Eisenbergiella | 19.60 | 2.12 | 25-26 |
| OTU_204 Bacteria;Firmicutes;Clostridia;Clostridiales;Lachnospiraceae;Tyzzerella 3;[Clostridium] colinum | 14.58 | -2.14 | 25-26 |
| OTU_128 Bacteria;Firmicutes;Clostridia;Clostridiales;Ruminococcaceae;[Eubacterium] coprostanoligenes group | 71.82 | 2.91 | 26-27 |
| OTU_398 Bacteria;Firmicutes;Clostridia;Clostridiales;Lachnospiraceae;Roseburia | 13.92 | 2.79 | 26-27 |
| OTU_85 Bacteria;Firmicutes;Clostridia;Clostridiales;Clostridiales vadinBB60 group | 782.32 | 2.68 | 26-27 |
| OTU_591 Bacteria;Firmicutes;Clostridia;Clostridiales;Clostridiales vadinBB60 group | 10.78 | 2.63 | 26-27 |
| OTU_680 Bacteria;Firmicutes;Clostridia;Clostridiales;Ruminococcaceae;Ruminococcaceae UCG-014 | 13.73 | 2.63 | 26-27 |
| OTU_1505 Bacteria;Firmicutes;Clostridia;Clostridiales;Ruminococcaceae;Ruminococcaceae UCG-014 | 9.09 | 2.48 | 26-27 |
| OTU_8121 Bacteria;Firmicutes;Clostridia;Clostridiales;Clostridiales vadinBB60 group | 299.08 | 2.53 | 26-27 |
| OTU_902 Bacteria;Firmicutes;Clostridia;Clostridiales;Clostridiales vadinBB60 group | 9.22 | 2.55 | 26-27 |
| OTU_526 Bacteria;Firmicutes;Clostridia;Clostridiales;Lachnospiraceae;Lachnoclostridium | 34.07 | 2.42 | 26-27 |
| OTU_231 Bacteria;Firmicutes;Clostridia;Clostridiales;Ruminococcaceae;Flavonifractor | 30.02 | 2.46 | 26-27 |
| OTU_1010 Bacteria;Firmicutes;Clostridia;Clostridiales;Lachnospiraceae | 9.02 | 2.33 | 26-27 |
| OTU_464 Bacteria;Firmicutes;Clostridia;Clostridiales;Ruminococcaceae;Ruminiclostridium 5 | 5.89 | 2.28 | 26-27 |
| OTU_332 Bacteria;Firmicutes;Clostridia;Clostridiales;Ruminococcaceae;Ruminococcaceae UCG-008 | 7.27 | 2.21 | 26-27 |
| OTU_162 Bacteria;Firmicutes;Clostridia;Clostridiales;Clostridiales vadinBB60 group | 8.07 | 2.07 | 26-27 |
| OTU_1098 Bacteria;Firmicutes;Clostridia;Clostridiales;Lachnospiraceae;Lachnospiraceae UCG-006 | 6.95 | 2.04 | 26-27 |
| OTU_192 Bacteria;Firmicutes;Clostridia;Clostridiales;Clostridiales vadinBB60 group | 35.57 | 2.16 | 27-28 |
| OTU_296 Bacteria;Firmicutes;Clostridia;Clostridiales;Peptostreptococcaceae;Intestinibacter | 24.92 | 2.19 | 27-28 |
| OTU_187 Bacteria;Firmicutes;Clostridia;Clostridiales;Ruminococcaceae;Ruminococcaceae UCG-014 | 30.56 | -3.16 | 27-28 |
| OTU_64 Bacteria;Firmicutes;Clostridia;Clostridiales;Ruminococcaceae;Ruminiclostridium 5 | 710.20 | -3.01 | 27-28 |
| OTU_591 Bacteria;Firmicutes;Clostridia;Clostridiales;Clostridiales vadinBB60 group | 10.79 | -2.72 | 27-28 |
| OTU_231 Bacteria;Firmicutes;Clostridia;Clostridiales;Ruminococcaceae;Flavonifractor | 29.68 | -2.71 | 27-28 |
| OTU_902 Bacteria;Firmicutes;Clostridia;Clostridiales;Clostridiales vadinBB60 group | 9.22 | -2.63 | 27-28 |
| OTU_1505 Bacteria;Firmicutes;Clostridia;Clostridiales;Ruminococcaceae;Ruminococcaceae UCG-014 | 9.36 | -2.34 | 27-28 |
| OTU_614 Bacteria;Tenericutes;Mollicutes;Mollicutes RF9 | 14.55 | -2.34 | 27-28 |
| OTU_1010 Bacteria;Firmicutes;Clostridia;Clostridiales;Lachnospiraceae | 9.09 | -2.33 | 27-28 |
| OTU_1098 Bacteria;Firmicutes;Clostridia;Clostridiales;Lachnospiraceae;Lachnospiraceae UCG-006 | 6.77 | -2.23 | 27-28 |
| OTU_6262 Bacteria;Firmicutes;Clostridia;Clostridiales;Ruminococcaceae;Ruminococcaceae UCG-014 | 431.75 | -2.13 | 27-28 |
| OTU_526 Bacteria;Firmicutes;Clostridia;Clostridiales;Lachnospiraceae;Lachnoclostridium | 35.09 | -2.12 | 27-28 |
| OTU_1399 Bacteria;Firmicutes;Clostridia;Clostridiales;Ruminococcaceae;Ruminococcaceae UCG-014 | 4.77 | -2.03 | 27-28 |
| OTU_286 Bacteria;Firmicutes;Clostridia;Clostridiales;Ruminococcaceae;[Eubacterium] coprostanoligenes group | 29.74 | 2.15 | 28-29 |
| OTU_231 Bacteria;Firmicutes;Clostridia;Clostridiales;Ruminococcaceae;Flavonifractor | 41.42 | 2.04 | 30-31 |
| OTU_95 Bacteria;Firmicutes;Clostridia;Clostridiales;Ruminococcaceae;Subdoligranulum | 81.73 | 5.92 | Day03-07-Day08-14 |
| OTU_248 Bacteria;Firmicutes;Clostridia;Clostridiales;Ruminococcaceae;Faecalibacterium | 43.97 | 5.71 | Day03-07-Day08-14 |
| OTU_155 Bacteria;Firmicutes;Clostridia;Clostridiales;Ruminococcaceae;Ruminococcaceae UCG-014 | 24.39 | 5.05 | Day03-07-Day08-14 |
| OTU_172 Bacteria;Firmicutes;Clostridia;Clostridiales;Ruminococcaceae;Ruminiclostridium 5 | 25.21 | 4.76 | Day03-07-Day08-14 |
| OTU_136 Bacteria;Proteobacteria;Gammaproteobacteria;Enterobacteriales;Enterobacteriaceae;Enterobacter | 217.12 | -5.40 | Day03-07-Day08-14 |
| OTU_195 Bacteria;Firmicutes;Clostridia;Clostridiales;Lachnospiraceae;Pseudobutyrivibrio | 72.67 | 4.13 | Day03-07-Day08-14 |
| OTU_1016 Bacteria;Firmicutes;Bacilli;Lactobacillales;Enterococcaceae;Enterococcus;unidentified marine bacterioplankton | 7.52 | -3.91 | Day03-07-Day08-14 |
| OTU_343 Bacteria;Firmicutes;Clostridia;Clostridiales;Ruminococcaceae;Ruminiclostridium 9 | 13.41 | 4.16 | Day03-07-Day08-14 |
| OTU_6332 Bacteria;Firmicutes;Clostridia;Clostridiales;Ruminococcaceae;Flavonifractor | 92.80 | -4.83 | Day03-07-Day08-14 |
| OTU_293 Bacteria;Firmicutes;Clostridia;Clostridiales;Clostridiales vadinBB60 group | 16.96 | 4.83 | Day03-07-Day08-14 |
| OTU_336 Bacteria;Firmicutes;Bacilli;Lactobacillales;Enterococcaceae;Enterococcus;Enterococcus faecium DO | 21.88 | -3.91 | Day03-07-Day08-14 |
| OTU_113 Bacteria;Firmicutes;Clostridia;Clostridiales;Ruminococcaceae;Flavonifractor | 299.35 | -5.09 | Day03-07-Day08-14 |
| OTU_4065 Bacteria;Firmicutes;Clostridia;Clostridiales;Ruminococcaceae;Subdoligranulum | 39.33 | 3.86 | Day03-07-Day08-14 |
| OTU_170 Bacteria;Firmicutes;Clostridia;Clostridiales;Ruminococcaceae;Ruminococcaceae UCG-014 | 24.40 | 3.96 | Day03-07-Day08-14 |
| OTU_34 Bacteria;Firmicutes;Clostridia;Clostridiales;Ruminococcaceae;Subdoligranulum | 158.74 | 4.41 | Day03-07-Day08-14 |
| OTU_275 Bacteria;Firmicutes;Clostridia;Clostridiales;Lachnospiraceae;Acetitomaculum | 6.90 | 3.30 | Day03-07-Day08-14 |
| OTU_12052 Bacteria;Proteobacteria;Gammaproteobacteria;Enterobacteriales;Enterobacteriaceae;Escherichia-Shigella | 21.11 | -2.74 | Day03-07-Day08-14 |
| OTU_11502 Bacteria;Proteobacteria;Gammaproteobacteria;Enterobacteriales;Enterobacteriaceae;Escherichia-Shigella;Shigella flexneri K-671 | 7.52 | -2.24 | Day03-07-Day08-14 |
| OTU_123 Bacteria;Actinobacteria;Coriobacteriia;Coriobacteriales;Coriobacteriaceae;Eggerthella | 104.91 | 3.16 | Day03-07-Day08-14 |
| OTU_3738 Bacteria;Proteobacteria;Gammaproteobacteria;Enterobacteriales;Enterobacteriaceae;Escherichia-Shigella | 47.68 | -2.83 | Day03-07-Day08-14 |
| OTU_298 Bacteria;Firmicutes;Clostridia;Clostridiales;Ruminococcaceae;Ruminiclostridium 9 | 11.11 | 2.82 | Day03-07-Day08-14 |
| OTU_31 Bacteria;Firmicutes;Clostridia;Clostridiales;Ruminococcaceae;Ruminococcaceae UCG-005 | 19.39 | 4.19 | Day03-07-Day08-14 |
| OTU_2587 Bacteria;Proteobacteria;Gammaproteobacteria;Enterobacteriales;Enterobacteriaceae;Escherichia-Shigella | 64.83 | -2.78 | Day03-07-Day08-14 |
| OTU_109 Bacteria;Firmicutes;Clostridia;Clostridiales;Ruminococcaceae;Ruminococcaceae UCG-014 | 35.09 | 4.06 | Day03-07-Day08-14 |
| OTU_13523 Bacteria;Firmicutes;Bacilli;Bacillales;Bacillaceae;Bacillus | 71.97 | 4.09 | Day03-07-Day08-14 |
| OTU_86 Bacteria;Firmicutes;Bacilli;Lactobacillales;Streptococcaceae;Streptococcus | 8.28 | -3.17 | Day03-07-Day08-14 |
| OTU_466 Bacteria;Firmicutes;Clostridia;Clostridiales;Ruminococcaceae;Anaerotruncus | 4.88 | 2.56 | Day03-07-Day08-14 |
| OTU_5864 Bacteria;Firmicutes;Clostridia;Clostridiales;Ruminococcaceae;Ruminococcaceae UCG-014 | 9.47 | 3.61 | Day03-07-Day08-14 |
| OTU_418 Bacteria;Firmicutes;Bacilli;Lactobacillales;Lactobacillaceae;Lactobacillus;Lactobacillus coleohominis | 4.54 | 2.79 | Day03-07-Day08-14 |
| OTU_179 Bacteria;Firmicutes;Clostridia;Clostridiales;Ruminococcaceae;Acetanaerobacterium;Acetanaerobacterium elongatum | 5.73 | 3.19 | Day03-07-Day08-14 |
| OTU_266 Bacteria;Firmicutes;Clostridia;Clostridiales;Ruminococcaceae;Ruminococcaceae UCG-002 | 8.23 | 3.20 | Day03-07-Day08-14 |
| OTU_11103 Bacteria;Firmicutes;Clostridia;Clostridiales;Lachnospiraceae;Eisenbergiella | 7.31 | -2.19 | Day03-07-Day08-14 |
| OTU_374 Bacteria;Firmicutes;Clostridia;Clostridiales;Ruminococcaceae | 8.76 | 3.61 | Day03-07-Day08-14 |
| OTU_12293 Bacteria;Firmicutes;Clostridia;Clostridiales;Ruminococcaceae;Ruminiclostridium 5 | 8.47 | 3.29 | Day03-07-Day08-14 |
| OTU_2701 Bacteria;Firmicutes;Clostridia;Clostridiales;Lachnospiraceae;Eisenbergiella | 39.36 | -4.00 | Day03-07-Day08-14 |
| OTU_327 Bacteria;Firmicutes;Clostridia;Clostridiales;Ruminococcaceae | 7.65 | 2.37 | Day03-07-Day08-14 |
| OTU_290 Bacteria;Firmicutes;Clostridia;Clostridiales;Family XIII;[Eubacterium] nodatum group | 7.78 | 2.67 | Day03-07-Day08-14 |
| OTU_99 Bacteria;Firmicutes;Clostridia;Clostridiales;Clostridiales vadinBB60 group | 39.47 | 3.76 | Day03-07-Day08-14 |
| OTU_16 Bacteria;Firmicutes;Clostridia;Clostridiales;Lachnospiraceae;Eisenbergiella | 48.58 | 3.80 | Day03-07-Day08-14 |
| OTU_92 Bacteria;Firmicutes;Clostridia;Clostridiales;Clostridiales vadinBB60 group | 17.26 | 3.67 | Day03-07-Day08-14 |
| OTU_198 Bacteria;Firmicutes;Clostridia;Clostridiales;Ruminococcaceae;Faecalibacterium | 8.28 | 3.38 | Day03-07-Day08-14 |
| OTU_477 NA;NA;NA;NA;NA;NA;NA | 3.91 | -2.94 | Day03-07-Day08-14 |
| OTU_507 Bacteria;Proteobacteria;Gammaproteobacteria;Enterobacteriales;Enterobacteriaceae;Cronobacter;Enterobacter sp. enrichment culture clone HSL2 | 3.97 | -2.64 | Day03-07-Day08-14 |
| OTU_1369 Bacteria;Firmicutes;Clostridia;Clostridiales;Lachnospiraceae;Eisenbergiella | 11.53 | -2.10 | Day03-07-Day08-14 |
| OTU_230 Bacteria;Firmicutes;Clostridia;Clostridiales;Ruminococcaceae;Ruminiclostridium 5 | 22.45 | 2.78 | Day03-07-Day08-14 |
| OTU_279 Bacteria;Firmicutes;Clostridia;Clostridiales;Ruminococcaceae;Ruminiclostridium 5 | 7.50 | 2.16 | Day03-07-Day08-14 |
| OTU_610 Bacteria;Firmicutes;Clostridia;Clostridiales;Ruminococcaceae;Anaerotruncus | 4.11 | 2.31 | Day03-07-Day08-14 |
| OTU_382 Bacteria;Firmicutes;Clostridia;Clostridiales;Christensenellaceae;Christensenellaceae R-7 group | 3.78 | 2.43 | Day03-07-Day08-14 |
| OTU_54 Bacteria;Firmicutes;Clostridia;Clostridiales;Ruminococcaceae;Ruminococcaceae UCG-014 | 373.84 | 4.15 | Day03-07-Day08-14 |
| OTU_3453 Bacteria;Firmicutes;Clostridia;Clostridiales;Lachnospiraceae;Tyzzerella | 122.69 | 3.52 | Day03-07-Day08-14 |
| OTU_286 Bacteria;Firmicutes;Clostridia;Clostridiales;Ruminococcaceae;[Eubacterium] coprostanoligenes group | 6.63 | 3.10 | Day03-07-Day08-14 |
| OTU_12120 Bacteria;Firmicutes;Clostridia;Clostridiales;Clostridiales vadinBB60 group | 6.75 | 3.08 | Day03-07-Day08-14 |
| OTU_6707 Bacteria;Firmicutes;Clostridia;Clostridiales;Ruminococcaceae | 21.36 | 3.60 | Day03-07-Day08-14 |
| OTU_305 Bacteria;Firmicutes;Clostridia;Clostridiales;Ruminococcaceae;Ruminococcaceae UCG-014 | 11.30 | 3.12 | Day03-07-Day08-14 |
| OTU_417 No blast hit | 5.16 | 2.84 | Day03-07-Day08-14 |
| OTU_138 Bacteria;Firmicutes;Bacilli;Lactobacillales;Lactobacillaceae;Lactobacillus;Lactobacillus salivarius GJ-24 | 32.04 | 2.92 | Day03-07-Day08-14 |
| OTU_2496 Bacteria;Firmicutes;Clostridia;Clostridiales;Lachnospiraceae;Tyzzerella | 8.43 | 3.02 | Day03-07-Day08-14 |
| OTU_80 Bacteria;Firmicutes;Clostridia;Clostridiales;Lachnospiraceae;Blautia | 251.36 | 3.23 | Day03-07-Day08-14 |
| OTU_119 Bacteria;Firmicutes;Clostridia;Clostridiales;Lachnospiraceae;Blautia | 138.97 | 2.84 | Day03-07-Day08-14 |
| OTU_180 Bacteria;Firmicutes;Clostridia;Clostridiales;Ruminococcaceae;Ruminococcus 1 | 8.05 | 2.89 | Day03-07-Day08-14 |
| OTU_105 Bacteria;Firmicutes;Clostridia;Clostridiales;Ruminococcaceae;Ruminococcaceae NK4A214 group | 5.21 | 2.95 | Day03-07-Day08-14 |
| OTU_686 No blast hit | 2.82 | -2.16 | Day03-07-Day08-14 |
| OTU_117 Bacteria;Firmicutes;Clostridia;Clostridiales;Ruminococcaceae | 83.04 | 2.93 | Day03-07-Day08-14 |
| OTU_178 Bacteria;Firmicutes;Clostridia;Clostridiales;Ruminococcaceae;Anaerotruncus | 11.14 | 3.04 | Day03-07-Day08-14 |
| OTU_217 Bacteria;Firmicutes;Clostridia;Clostridiales;Family XIII;Family XIII UCG-001 | 3.71 | 2.37 | Day03-07-Day08-14 |
| OTU_235 Bacteria;Firmicutes;Clostridia;Clostridiales;Ruminococcaceae;Anaerotruncus | 7.07 | 2.80 | Day03-07-Day08-14 |
| OTU_67 Bacteria;Firmicutes;Clostridia;Clostridiales;Ruminococcaceae;Ruminococcaceae NK4A214 group | 32.67 | 3.20 | Day03-07-Day08-14 |
| OTU_83 Bacteria;Firmicutes;Clostridia;Clostridiales;Ruminococcaceae;Ruminococcus 1 | 5.08 | 3.00 | Day03-07-Day08-14 |
| OTU_454 No blast hit | 7.13 | 2.29 | Day03-07-Day08-14 |
| OTU_285 Bacteria;Firmicutes;Clostridia;Clostridiales;Lachnospiraceae;Roseburia | 3.57 | 2.43 | Day03-07-Day08-14 |
| OTU_15571 Bacteria;Firmicutes;Clostridia;Clostridiales;Ruminococcaceae | 5.12 | 2.62 | Day03-07-Day08-14 |
| OTU_1047 Bacteria;Firmicutes;Clostridia;Clostridiales;Ruminococcaceae | 3.80 | 2.53 | Day03-07-Day08-14 |
| OTU_73 Bacteria;Firmicutes;Bacilli;Bacillales;Bacillaceae;Bacillus | 108.30 | 3.58 | Day03-07-Day08-14 |
| OTU_59 Bacteria;Firmicutes;Clostridia;Clostridiales;Ruminococcaceae;Ruminococcaceae UCG-005 | 394.86 | 3.73 | Day03-07-Day08-14 |
| OTU_3700 Bacteria;Firmicutes;Clostridia;Clostridiales;Defluviitaleaceae;Defluviitaleaceae UCG-011 | 41.17 | 2.79 | Day03-07-Day08-14 |
| OTU_126 Bacteria;Firmicutes;Clostridia;Clostridiales;Ruminococcaceae;Ruminococcaceae NK4A214 group | 10.01 | 2.81 | Day03-07-Day08-14 |
| OTU_132 Bacteria;Firmicutes;Clostridia;Clostridiales;Ruminococcaceae;Ruminiclostridium 5 | 16.50 | 2.80 | Day03-07-Day08-14 |
| OTU_35 Bacteria;Firmicutes;Clostridia;Clostridiales;Clostridiales vadinBB60 group | 529.57 | 3.05 | Day03-07-Day08-14 |
| OTU_370 No blast hit | 10.54 | 2.16 | Day03-07-Day08-14 |
| OTU_1 Bacteria;Firmicutes;Clostridia;Clostridiales;Ruminococcaceae;Faecalibacterium | 526.62 | 3.93 | Day03-07-Day08-14 |
| OTU_3296 Bacteria;Firmicutes;Clostridia;Clostridiales;Ruminococcaceae | 7.38 | 2.07 | Day03-07-Day08-14 |
| OTU_13602 Bacteria;Firmicutes;Clostridia;Clostridiales;Clostridiales vadinBB60 group | 4.85 | 2.72 | Day03-07-Day08-14 |
| OTU_13 Bacteria;Firmicutes;Clostridia;Clostridiales;Lachnospiraceae;Tyzzerella | 338.74 | 3.23 | Day03-07-Day08-14 |
| OTU_360 Bacteria;Firmicutes;Clostridia;Clostridiales;Ruminococcaceae;Ruminococcus 2 | 3.97 | 2.31 | Day03-07-Day08-14 |
| OTU_414 No blast hit | 8.64 | 2.14 | Day03-07-Day08-14 |
| OTU_48 Bacteria;Firmicutes;Clostridia;Clostridiales;Defluviitaleaceae;Defluviitaleaceae UCG-011 | 101.45 | 2.81 | Day03-07-Day08-14 |
| OTU_218 Bacteria;Firmicutes;Clostridia;Clostridiales;Ruminococcaceae;Butyricicoccus;Butyricicoccus pullicaecorum 1.2 | 6.60 | 2.63 | Day03-07-Day08-14 |
| OTU_242 Bacteria;Firmicutes;Clostridia;Clostridiales;Ruminococcaceae;Anaerotruncus | 2.88 | 2.07 | Day03-07-Day08-14 |
| OTU_127 Bacteria;Firmicutes;Clostridia;Clostridiales;Ruminococcaceae;Ruminiclostridium 9 | 41.18 | 2.37 | Day03-07-Day08-14 |
| OTU_267 Bacteria;Actinobacteria;Coriobacteriia;Coriobacteriales;Coriobacteriaceae | 3.16 | 2.23 | Day03-07-Day08-14 |
| OTU_234 Bacteria;Firmicutes;Erysipelotrichia;Erysipelotrichales;Erysipelotrichaceae | 10.21 | 2.00 | Day03-07-Day08-14 |
| OTU_319 No blast hit | 9.23 | 2.24 | Day03-07-Day08-14 |
| OTU_3390 Bacteria;Firmicutes;Clostridia;Clostridiales;Ruminococcaceae;[Eubacterium] coprostanoligenes group | 212.08 | 3.05 | Day03-07-Day08-14 |
| OTU_37 Bacteria;Firmicutes;Clostridia;Clostridiales;Clostridiales vadinBB60 group | 3.76 | 2.51 | Day03-07-Day08-14 |
| OTU_64 Bacteria;Firmicutes;Clostridia;Clostridiales;Ruminococcaceae;Ruminiclostridium 5 | 7.52 | 2.70 | Day03-07-Day08-14 |
| OTU_3027 Bacteria;Firmicutes;Clostridia;Clostridiales;Ruminococcaceae;Intestinimonas | 16.05 | 2.80 | Day03-07-Day08-14 |
| OTU_149 Bacteria;Firmicutes;Clostridia;Clostridiales;Lachnospiraceae;Lachnoclostridium | 10.91 | 2.39 | Day03-07-Day08-14 |
| OTU_9 Bacteria;Firmicutes;Negativicutes;Selenomonadales;Veillonellaceae;Megamonas | 3.26 | 2.28 | Day03-07-Day08-14 |
| OTU_314 Bacteria;Firmicutes;Clostridia;Clostridiales;Ruminococcaceae | 2.92 | 2.06 | Day03-07-Day08-14 |
| OTU_68 Bacteria;Firmicutes;Clostridia;Clostridiales;Ruminococcaceae;Hydrogenoanaerobacterium | 3.48 | 2.39 | Day03-07-Day08-14 |
| OTU_148 Bacteria;Firmicutes;Clostridia;Clostridiales;Clostridiales vadinBB60 group | 48.86 | 2.87 | Day03-07-Day08-14 |
| OTU_301 Bacteria;Firmicutes;Clostridia;Clostridiales;Ruminococcaceae;Ruminiclostridium 5 | 16.18 | 2.15 | Day03-07-Day08-14 |
| OTU_5053 Bacteria;Firmicutes;Clostridia;Clostridiales;Lachnospiraceae | 21.53 | 2.42 | Day03-07-Day08-14 |
| OTU_541 Bacteria;Firmicutes;Clostridia;Clostridiales;Ruminococcaceae;Ruminococcaceae UCG-013 | 21.29 | 2.06 | Day03-07-Day08-14 |
| OTU_6463 Bacteria;Firmicutes;Clostridia;Clostridiales;Ruminococcaceae;Ruminococcaceae NK4A214 group | 11.11 | 2.29 | Day03-07-Day08-14 |
| OTU_358 Bacteria;Firmicutes;Clostridia;Clostridiales;Ruminococcaceae | 8.39 | 2.09 | Day03-07-Day08-14 |
| OTU_243 Bacteria;Firmicutes;Clostridia;Clostridiales;Clostridiales vadinBB60 group | 38.69 | 2.85 | Day03-07-Day08-14 |
| OTU_220 Bacteria;Firmicutes;Clostridia;Clostridiales;Ruminococcaceae;Ruminococcaceae UCG-005 | 34.91 | 2.23 | Day03-07-Day08-14 |
| OTU_778 Bacteria;Firmicutes;Clostridia;Clostridiales;Ruminococcaceae;Ruminococcaceae UCG-014 | 4.12 | 2.18 | Day03-07-Day08-14 |
| OTU_21 Bacteria;Firmicutes;Clostridia;Clostridiales;Ruminococcaceae | 10.68 | 2.49 | Day03-07-Day08-14 |
| OTU_526 Bacteria;Firmicutes;Clostridia;Clostridiales;Lachnospiraceae;Lachnoclostridium | 7.88 | 2.17 | Day03-07-Day08-14 |
| OTU_167 Bacteria;Firmicutes;Clostridia;Clostridiales;Lachnospiraceae;Shuttleworthia | 30.99 | 2.31 | Day03-07-Day08-14 |
| OTU_185 Bacteria;Firmicutes;Clostridia;Clostridiales;Ruminococcaceae;Ruminiclostridium 5 | 5.43 | 2.08 | Day03-07-Day08-14 |
| OTU_42 Bacteria;Firmicutes;Clostridia;Clostridiales;Clostridiales vadinBB60 group | 5.73 | 2.42 | Day03-07-Day08-14 |
| OTU_36 Bacteria;Firmicutes;Clostridia;Clostridiales;Ruminococcaceae;[Eubacterium] coprostanoligenes group | 378.13 | 2.82 | Day03-07-Day08-14 |
| OTU_101 Bacteria;Firmicutes;Clostridia;Clostridiales;Ruminococcaceae;Anaerotruncus | 2.98 | 2.13 | Day03-07-Day08-14 |
| OTU_130 Bacteria;Firmicutes;Clostridia;Clostridiales;Ruminococcaceae;Ruminococcaceae UCG-005 | 9.26 | 2.24 | Day03-07-Day08-14 |
| OTU_14105 Bacteria;Firmicutes;Clostridia;Clostridiales;Ruminococcaceae;Ruminococcaceae UCG-009 | 250.90 | 2.23 | Day03-07-Day08-14 |
| OTU_43 Bacteria;Actinobacteria;Actinobacteria;Bifidobacteriales;Bifidobacteriaceae;Bifidobacterium;Bifidobacterium saeculare | 4.58 | 2.15 | Day03-07-Day08-14 |
| OTU_6238 Bacteria;Actinobacteria;Actinobacteria;Bifidobacteriales;Bifidobacteriaceae;Bifidobacterium;Bifidobacterium saeculare | 3.20 | 2.00 | Day03-07-Day08-14 |
| OTU_79 Bacteria;Firmicutes;Clostridia;Clostridiales;Clostridiales vadinBB60 group | 2.87 | 2.07 | Day03-07-Day08-14 |
| OTU_108 Bacteria;Firmicutes;Clostridia;Clostridiales;Ruminococcaceae;Anaerofilum | 48.59 | 2.53 | Day03-07-Day08-14 |
| OTU_1190 Bacteria;Firmicutes;Clostridia;Clostridiales;Ruminococcaceae;Ruminiclostridium;Clostridiales bacterium DJF_B152 | 4.01 | 2.06 | Day03-07-Day08-14 |
| OTU_88 Bacteria;Firmicutes;Clostridia;Clostridiales;Lachnospiraceae;Lachnospiraceae FE2018 group | 174.59 | 2.33 | Day03-07-Day08-14 |
| OTU_605 Bacteria;Firmicutes;Clostridia;Clostridiales;Ruminococcaceae;Ruminococcaceae UCG-014 | 14.53 | 2.06 | Day03-07-Day08-14 |
| OTU_4425 Bacteria;Firmicutes;Clostridia;Clostridiales;Clostridiales vadinBB60 group | 93.57 | 2.53 | Day03-07-Day08-14 |
| OTU_3463 Bacteria;Firmicutes;Clostridia;Clostridiales;Ruminococcaceae | 7.95 | 2.06 | Day03-07-Day08-14 |
| OTU_380 Bacteria;Firmicutes;Clostridia;Clostridiales;Clostridiales vadinBB60 group | 35.61 | 2.06 | Day03-07-Day08-14 |
| OTU_315 Bacteria;Firmicutes;Clostridia;Clostridiales;Lachnospiraceae | 38.25 | -2.20 | Day03-07-Day08-14 |
| OTU_53 Bacteria;Firmicutes;Clostridia;Clostridiales;Ruminococcaceae;Ruminiclostridium 5 | 75.08 | 2.03 | Day03-07-Day08-14 |
| OTU_1729 Bacteria;Firmicutes;Clostridia;Clostridiales;Clostridiales vadinBB60 group | 539.18 | 2.68 | Day03-07-Day08-14 |
| OTU_14 Bacteria;Firmicutes;Clostridia;Clostridiales;Clostridiales vadinBB60 group | 1344.43 | 2.73 | Day03-07-Day08-14 |
| OTU_90 Bacteria;Firmicutes;Clostridia;Clostridiales;Lachnospiraceae | 310.12 | 2.09 | Day03-07-Day08-14 |
| OTU_1024 Bacteria;Firmicutes;Clostridia;Clostridiales;Ruminococcaceae;Faecalibacterium | 637.20 | 2.02 | Day03-07-Day08-14 |
| OTU_86 Bacteria;Firmicutes;Bacilli;Lactobacillales;Streptococcaceae;Streptococcus | 146.99 | 6.84 | Day08-14-Day15-24 |
| OTU_70 Bacteria;Firmicutes;Clostridia;Clostridiales;Clostridiales vadinBB60 group | 131.80 | 7.61 | Day08-14-Day15-24 |
| OTU_82 Bacteria;Firmicutes;Clostridia;Clostridiales;Ruminococcaceae;[Eubacterium] coprostanoligenes group | 204.30 | 8.37 | Day08-14-Day15-24 |
| OTU_75 Bacteria;Firmicutes;Clostridia;Clostridiales;Ruminococcaceae;Ruminococcaceae UCG-014 | 213.10 | 8.11 | Day08-14-Day15-24 |
| OTU_3674 Bacteria;Firmicutes;Clostridia;Clostridiales;Clostridiales vadinBB60 group | 96.35 | 7.18 | Day08-14-Day15-24 |
| OTU_94 Bacteria;Firmicutes;Clostridia;Clostridiales;Ruminococcaceae;Ruminococcaceae UCG-014 | 65.43 | 6.19 | Day08-14-Day15-24 |
| OTU_97 Bacteria;Firmicutes;Clostridia;Clostridiales;Christensenellaceae;Christensenellaceae R-7 group | 67.16 | 6.27 | Day08-14-Day15-24 |
| OTU_7 Bacteria;Firmicutes;Clostridia;Clostridiales;Ruminococcaceae | 132.53 | 7.44 | Day08-14-Day15-24 |
| OTU_199 Bacteria;Firmicutes;Clostridia;Clostridiales;Christensenellaceae;Christensenellaceae R-7 group | 41.01 | 5.24 | Day08-14-Day15-24 |
| OTU_102 Bacteria;Firmicutes;Clostridia;Clostridiales;Ruminococcaceae;Ruminiclostridium 9 | 74.23 | 6.48 | Day08-14-Day15-24 |
| OTU_207 Bacteria;Firmicutes;Clostridia;Clostridiales;Ruminococcaceae;Ruminococcaceae UCG-014 | 41.70 | 5.76 | Day08-14-Day15-24 |
| OTU_116 Bacteria;Firmicutes;Clostridia;Clostridiales;Clostridiales vadinBB60 group | 140.11 | 5.88 | Day08-14-Day15-24 |
| OTU_137 Bacteria;Firmicutes;Clostridia;Clostridiales;Clostridiales vadinBB60 group | 47.10 | 6.14 | Day08-14-Day15-24 |
| OTU_6238 Bacteria;Actinobacteria;Actinobacteria;Bifidobacteriales;Bifidobacteriaceae;Bifidobacterium;Bifidobacterium saeculare | 160.11 | 5.64 | Day08-14-Day15-24 |
| OTU_101 Bacteria;Firmicutes;Clostridia;Clostridiales;Ruminococcaceae;Anaerotruncus | 159.98 | 5.74 | Day08-14-Day15-24 |
| OTU_114 Bacteria;Tenericutes;Mollicutes;Mollicutes RF9 | 53.67 | 6.44 | Day08-14-Day15-24 |
| OTU_63 Bacteria;Firmicutes;Clostridia;Clostridiales;Ruminococcaceae;Ruminococcaceae UCG-014 | 115.45 | 5.83 | Day08-14-Day15-24 |
| OTU_142 Bacteria;Firmicutes;Clostridia;Clostridiales;Ruminococcaceae;Ruminococcaceae UCG-010 | 42.68 | 5.53 | Day08-14-Day15-24 |
| OTU_214 Bacteria;Firmicutes;Clostridia;Clostridiales;Lachnospiraceae | 116.69 | 6.62 | Day08-14-Day15-24 |
| OTU_140 Bacteria;Firmicutes;Clostridia;Clostridiales;Ruminococcaceae;Ruminococcaceae UCG-004 | 20.32 | 4.52 | Day08-14-Day15-24 |
| OTU_1938 Bacteria;Firmicutes;Clostridia;Clostridiales;Ruminococcaceae | 78.43 | 6.48 | Day08-14-Day15-24 |
| OTU_154 Bacteria;Firmicutes;Clostridia;Clostridiales;Ruminococcaceae;Ruminiclostridium 5 | 95.76 | 5.25 | Day08-14-Day15-24 |
| OTU_43 Bacteria;Actinobacteria;Actinobacteria;Bifidobacteriales;Bifidobacteriaceae;Bifidobacterium;Bifidobacterium saeculare | 410.24 | 5.72 | Day08-14-Day15-24 |
| OTU_111 Bacteria;Firmicutes;Clostridia;Clostridiales;Ruminococcaceae;Ruminococcaceae UCG-014 | 37.58 | 5.92 | Day08-14-Day15-24 |
| OTU_6396 Bacteria;Firmicutes;Clostridia;Clostridiales;Clostridiales vadinBB60 group | 64.58 | 5.88 | Day08-14-Day15-24 |
| OTU_55 Bacteria;Firmicutes;Clostridia;Clostridiales;Ruminococcaceae;Ruminococcaceae UCG-014 | 438.98 | 6.96 | Day08-14-Day15-24 |
| OTU_173 Bacteria;Firmicutes;Clostridia;Clostridiales;Ruminococcaceae;Anaerotruncus | 47.69 | 4.95 | Day08-14-Day15-24 |
| OTU_151 Bacteria;Firmicutes;Clostridia;Clostridiales;Ruminococcaceae;Anaerotruncus | 32.42 | 5.66 | Day08-14-Day15-24 |
| OTU_84 Bacteria;Firmicutes;Clostridia;Clostridiales;Lachnospiraceae | 142.18 | 5.44 | Day08-14-Day15-24 |
| OTU_156 Bacteria;Firmicutes;Clostridia;Clostridiales;Ruminococcaceae;Oscillospira | 31.57 | 5.18 | Day08-14-Day15-24 |
| OTU_66 Bacteria;Firmicutes;Clostridia;Clostridiales;Ruminococcaceae | 43.21 | 5.76 | Day08-14-Day15-24 |
| OTU_6262 Bacteria;Firmicutes;Clostridia;Clostridiales;Ruminococcaceae;Ruminococcaceae UCG-014 | 268.68 | 5.27 | Day08-14-Day15-24 |
| OTU_87 Bacteria;Firmicutes;Clostridia;Clostridiales;Ruminococcaceae;Ruminococcaceae UCG-014 | 128.64 | 5.16 | Day08-14-Day15-24 |
| OTU_118 Bacteria;Firmicutes;Clostridia;Clostridiales;Clostridiales vadinBB60 group | 54.77 | 5.99 | Day08-14-Day15-24 |
| OTU_7674 Bacteria;Tenericutes;Mollicutes;Mollicutes RF9 | 26.95 | 5.43 | Day08-14-Day15-24 |
| OTU_191 Bacteria;Firmicutes;Clostridia;Clostridiales;Ruminococcaceae;Ruminococcaceae UCG-014 | 25.65 | 4.22 | Day08-14-Day15-24 |
| OTU_228 Bacteria;Firmicutes;Clostridia;Clostridiales;Ruminococcaceae;Ruminococcaceae UCG-014 | 20.31 | 5.02 | Day08-14-Day15-24 |
| OTU_233 Bacteria;Firmicutes;Clostridia;Clostridiales;Ruminococcaceae;Ruminiclostridium 1 | 16.34 | 4.43 | Day08-14-Day15-24 |
| OTU_15527 Bacteria;Firmicutes;Clostridia;Clostridiales;Ruminococcaceae;Ruminococcaceae UCG-014 | 20.71 | 5.04 | Day08-14-Day15-24 |
| OTU_124 Bacteria;Firmicutes;Clostridia;Clostridiales;Clostridiales vadinBB60 group | 79.40 | 4.90 | Day08-14-Day15-24 |
| OTU_105 Bacteria;Firmicutes;Clostridia;Clostridiales;Ruminococcaceae;Ruminococcaceae NK4A214 group | 101.24 | 4.19 | Day08-14-Day15-24 |
| OTU_159 Bacteria;Firmicutes;Clostridia;Clostridiales;Ruminococcaceae;Intestinimonas | 100.19 | 4.93 | Day08-14-Day15-24 |
| OTU_5826 Bacteria;Firmicutes;Clostridia;Clostridiales;Ruminococcaceae | 22.74 | 5.04 | Day08-14-Day15-24 |
| OTU_8131 Bacteria;Firmicutes;Clostridia;Clostridiales;Ruminococcaceae;Anaerotruncus | 58.18 | 4.54 | Day08-14-Day15-24 |
| OTU_213 Bacteria;Firmicutes;Clostridia;Clostridiales;Clostridiales vadinBB60 group | 19.70 | 4.97 | Day08-14-Day15-24 |
| OTU_6791 Bacteria;Firmicutes;Clostridia;Clostridiales;Ruminococcaceae;Ruminococcaceae UCG-004 | 8.57 | 3.54 | Day08-14-Day15-24 |
| OTU_169 Bacteria;Firmicutes;Clostridia;Clostridiales;Lachnospiraceae | 22.49 | 5.08 | Day08-14-Day15-24 |
| OTU_179 Bacteria;Firmicutes;Clostridia;Clostridiales;Ruminococcaceae;Acetanaerobacterium;Acetanaerobacterium elongatum | 52.84 | 3.14 | Day08-14-Day15-24 |
| OTU_254 Bacteria;Firmicutes;Clostridia;Clostridiales;Christensenellaceae;Christensenellaceae R-7 group | 19.07 | 3.45 | Day08-14-Day15-24 |
| OTU_438 Bacteria;Firmicutes;Clostridia;Clostridiales;Ruminococcaceae;Ruminiclostridium | 27.85 | 4.88 | Day08-14-Day15-24 |
| OTU_184 Bacteria;Firmicutes;Clostridia;Clostridiales;Ruminococcaceae;Ruminococcaceae UCG-014 | 16.28 | 4.20 | Day08-14-Day15-24 |
| OTU_263 Bacteria;Firmicutes;Clostridia;Clostridiales;Ruminococcaceae;Anaerotruncus | 14.70 | 4.52 | Day08-14-Day15-24 |
| OTU_249 Bacteria;Firmicutes;Clostridia;Clostridiales;Ruminococcaceae;Anaerofilum | 9.72 | 3.88 | Day08-14-Day15-24 |
| OTU_7826 Bacteria;Firmicutes;Clostridia;Clostridiales;Ruminococcaceae;Ruminococcaceae UCG-014 | 90.77 | 4.58 | Day08-14-Day15-24 |
| OTU_130 Bacteria;Firmicutes;Clostridia;Clostridiales;Ruminococcaceae;Ruminococcaceae UCG-005 | 96.14 | 3.42 | Day08-14-Day15-24 |
| OTU_14407 Bacteria;Firmicutes;Negativicutes;Selenomonadales;Veillonellaceae;Megamonas;unidentified | 132.33 | 5.81 | Day08-14-Day15-24 |
| OTU_162 Bacteria;Firmicutes;Clostridia;Clostridiales;Clostridiales vadinBB60 group | 22.40 | 5.05 | Day08-14-Day15-24 |
| OTU_369 Bacteria;Tenericutes;Mollicutes;Mollicutes RF9 | 10.35 | 3.69 | Day08-14-Day15-24 |
| OTU_201 Bacteria;Firmicutes;Clostridia;Clostridiales;Clostridiales vadinBB60 group | 14.31 | 4.48 | Day08-14-Day15-24 |
| OTU_74 Bacteria;Firmicutes;Clostridia;Clostridiales;Ruminococcaceae;Ruminococcaceae UCG-014 | 106.89 | 3.98 | Day08-14-Day15-24 |
| OTU_602 Bacteria;Firmicutes;Clostridia;Clostridiales;Ruminococcaceae;Ruminococcaceae UCG-014 | 35.39 | 4.55 | Day08-14-Day15-24 |
| OTU_13399 Bacteria;Firmicutes;Clostridia;Clostridiales;Ruminococcaceae;Ruminococcaceae UCG-002 | 18.11 | 3.79 | Day08-14-Day15-24 |
| OTU_292 Bacteria;Firmicutes;Clostridia;Clostridiales;Peptococcaceae | 10.58 | 3.41 | Day08-14-Day15-24 |
| OTU_229 Bacteria;Firmicutes;Clostridia;Clostridiales;Clostridiales vadinBB60 group | 13.21 | 4.38 | Day08-14-Day15-24 |
| OTU_283 Bacteria;Firmicutes;Clostridia;Clostridiales;Clostridiales vadinBB60 group | 9.75 | 3.86 | Day08-14-Day15-24 |
| OTU_100 Bacteria;Firmicutes;Clostridia;Clostridiales;Clostridiales vadinBB60 group | 76.00 | 4.46 | Day08-14-Day15-24 |
| OTU_9 Bacteria;Firmicutes;Negativicutes;Selenomonadales;Veillonellaceae;Megamonas | 142.36 | 5.55 | Day08-14-Day15-24 |
| OTU_37 Bacteria;Firmicutes;Clostridia;Clostridiales;Clostridiales vadinBB60 group | 124.49 | 5.12 | Day08-14-Day15-24 |
| OTU_312 Bacteria;Firmicutes;Clostridia;Clostridiales;Lachnospiraceae | 11.39 | 4.16 | Day08-14-Day15-24 |
| OTU_15345 Bacteria;Firmicutes;Clostridia;Clostridiales;Clostridiales vadinBB60 group | 10.57 | 4.05 | Day08-14-Day15-24 |
| OTU_250 Bacteria;Firmicutes;Clostridia;Clostridiales;Ruminococcaceae | 8.07 | 3.47 | Day08-14-Day15-24 |
| OTU_399 Bacteria;Firmicutes;Clostridia;Clostridiales;Clostridiales vadinBB60 group | 12.76 | 3.95 | Day08-14-Day15-24 |
| OTU_552 Bacteria;Firmicutes;Clostridia;Clostridiales;Ruminococcaceae | 21.66 | 3.88 | Day08-14-Day15-24 |
| OTU_209 Bacteria;Firmicutes;Clostridia;Clostridiales;Clostridiales vadinBB60 group | 13.63 | 3.77 | Day08-14-Day15-24 |
| OTU_630 Bacteria;Firmicutes;Clostridia;Clostridiales;Ruminococcaceae;Intestinimonas | 12.89 | 3.64 | Day08-14-Day15-24 |
| OTU_242 Bacteria;Firmicutes;Clostridia;Clostridiales;Ruminococcaceae;Anaerotruncus | 24.98 | 3.17 | Day08-14-Day15-24 |
| OTU_6645 Bacteria;Firmicutes;Clostridia;Clostridiales;Clostridiales vadinBB60 group | 11.08 | 4.12 | Day08-14-Day15-24 |
| OTU_241 Bacteria;Firmicutes;Clostridia;Clostridiales;Ruminococcaceae;Ruminococcaceae V9D2013 group | 12.69 | 3.40 | Day08-14-Day15-24 |
| OTU_368 Bacteria;Firmicutes;Clostridia;Clostridiales;Clostridiaceae 1;Candidatus Arthromitus | 9.66 | -2.54 | Day08-14-Day15-24 |
| OTU_135 Bacteria;Firmicutes;Clostridia;Clostridiales;Ruminococcaceae;Ruminococcaceae NK4A214 group | 9.60 | 3.86 | Day08-14-Day15-24 |
| OTU_208 Bacteria;Firmicutes;Clostridia;Clostridiales;Ruminococcaceae;Ruminococcaceae UCG-014 | 18.06 | 3.22 | Day08-14-Day15-24 |
| OTU_389 Bacteria;Tenericutes;Mollicutes;Mollicutes RF9 | 8.64 | 2.93 | Day08-14-Day15-24 |
| OTU_240 Bacteria;Firmicutes;Clostridia;Clostridiales;Ruminococcaceae;Ruminococcaceae UCG-014 | 15.05 | 3.26 | Day08-14-Day15-24 |
| OTU_329 Bacteria;Tenericutes;Mollicutes;Mollicutes RF9 | 6.40 | 3.11 | Day08-14-Day15-24 |
| OTU_373 Bacteria;Firmicutes;Clostridia;Clostridiales;Ruminococcaceae;Ruminococcaceae UCG-010 | 6.91 | 3.11 | Day08-14-Day15-24 |
| OTU_328 Bacteria;Firmicutes;Clostridia;Clostridiales;Lachnospiraceae;Blautia | 20.30 | -2.76 | Day08-14-Day15-24 |
| OTU_149 Bacteria;Firmicutes;Clostridia;Clostridiales;Lachnospiraceae;Lachnoclostridium | 137.68 | 3.67 | Day08-14-Day15-24 |
| OTU_346 Bacteria;Firmicutes;Clostridia;Clostridiales;Lachnospiraceae;Lachnospiraceae UCG-010 | 6.55 | 2.35 | Day08-14-Day15-24 |
| OTU_126 Bacteria;Firmicutes;Clostridia;Clostridiales;Ruminococcaceae;Ruminococcaceae NK4A214 group | 81.97 | 2.99 | Day08-14-Day15-24 |
| OTU_359 Bacteria;Firmicutes;Clostridia;Clostridiales;Lachnospiraceae;Marvinbryantia | 7.61 | 3.01 | Day08-14-Day15-24 |
| OTU_113 Bacteria;Firmicutes;Clostridia;Clostridiales;Ruminococcaceae;Flavonifractor | 8.98 | -3.23 | Day08-14-Day15-24 |
| OTU_237 Bacteria;Firmicutes;Clostridia;Clostridiales;Ruminococcaceae;Anaerotruncus | 17.34 | 2.81 | Day08-14-Day15-24 |
| OTU_225 Bacteria;Firmicutes;Clostridia;Clostridiales;Clostridiales vadinBB60 group | 43.80 | 3.44 | Day08-14-Day15-24 |
| OTU_152 Bacteria;Firmicutes;Clostridia;Clostridiales;Ruminococcaceae;Ruminococcaceae UCG-014 | 65.87 | 3.73 | Day08-14-Day15-24 |
| OTU_83 Bacteria;Firmicutes;Clostridia;Clostridiales;Ruminococcaceae;Ruminococcus 1 | 198.87 | 3.72 | Day08-14-Day15-24 |
| OTU_287 Bacteria;Firmicutes;Clostridia;Clostridiales;Ruminococcaceae;Ruminococcaceae UCG-014 | 9.93 | 3.36 | Day08-14-Day15-24 |
| OTU_197 Bacteria;Firmicutes;Erysipelotrichia;Erysipelotrichales;Erysipelotrichaceae;Erysipelatoclostridium;bacterium ic1391 | 63.37 | 2.83 | Day08-14-Day15-24 |
| OTU_622 Bacteria;Actinobacteria;Actinobacteria;Corynebacteriales;Corynebacteriaceae;Corynebacterium 1;Corynebacterium glutamicum | 3.64 | -2.20 | Day08-14-Day15-24 |
| OTU_371 Bacteria;Firmicutes;Clostridia;Clostridiales;Lachnospiraceae;Lachnospiraceae NK4A136 group | 5.52 | 2.36 | Day08-14-Day15-24 |
| OTU_76 Bacteria;Firmicutes;Clostridia;Clostridiales;Ruminococcaceae;Ruminococcaceae UCG-014 | 7.77 | 3.58 | Day08-14-Day15-24 |
| OTU_386 Bacteria;Firmicutes;Clostridia;Clostridiales;Caldicoprobacteraceae;Caldicoprobacter | 5.64 | 3.09 | Day08-14-Day15-24 |
| OTU_193 Bacteria;Firmicutes;Clostridia;Clostridiales;Clostridiales vadinBB60 group | 7.05 | 3.39 | Day08-14-Day15-24 |
| OTU_16519 Bacteria;Firmicutes;Clostridia;Clostridiales;Ruminococcaceae | 3.22 | -2.17 | Day08-14-Day15-24 |
| OTU_153 Bacteria;Firmicutes;Clostridia;Clostridiales;Lachnospiraceae;[Eubacterium] oxidoreducens group | 9.27 | 3.58 | Day08-14-Day15-24 |
| OTU_222 Bacteria;Firmicutes;Clostridia;Clostridiales;Ruminococcaceae | 11.37 | 2.94 | Day08-14-Day15-24 |
| OTU_284 Bacteria;Firmicutes;Clostridia;Clostridiales;Ruminococcaceae;Anaerotruncus | 10.44 | 2.70 | Day08-14-Day15-24 |
| OTU_270 Bacteria;Firmicutes;Clostridia;Clostridiales;Ruminococcaceae;Anaerotruncus | 5.73 | 2.82 | Day08-14-Day15-24 |
| OTU_12120 Bacteria;Firmicutes;Clostridia;Clostridiales;Clostridiales vadinBB60 group | 54.10 | 2.92 | Day08-14-Day15-24 |
| OTU_6463 Bacteria;Firmicutes;Clostridia;Clostridiales;Ruminococcaceae;Ruminococcaceae NK4A214 group | 66.64 | 2.56 | Day08-14-Day15-24 |
| OTU_180 Bacteria;Firmicutes;Clostridia;Clostridiales;Ruminococcaceae;Ruminococcus 1 | 57.87 | 2.77 | Day08-14-Day15-24 |
| OTU_2511 Bacteria;Actinobacteria;Actinobacteria;Bifidobacteriales;Bifidobacteriaceae;Bifidobacterium;Bifidobacterium saeculare | 6.48 | 2.90 | Day08-14-Day15-24 |
| OTU_2496 Bacteria;Firmicutes;Clostridia;Clostridiales;Lachnospiraceae;Tyzzerella | 53.00 | 2.55 | Day08-14-Day15-24 |
| OTU_295 No blast hit | 9.62 | -2.60 | Day08-14-Day15-24 |
| OTU_388 Bacteria;Firmicutes;Clostridia;Clostridiales;Peptococcaceae | 4.74 | 2.82 | Day08-14-Day15-24 |
| OTU_204 Bacteria;Firmicutes;Clostridia;Clostridiales;Lachnospiraceae;Tyzzerella 3;[Clostridium] colinum | 9.45 | 3.33 | Day08-14-Day15-24 |
| OTU_510 Bacteria;Firmicutes;Clostridia;Clostridiales;Ruminococcaceae;Ruminococcaceae NK4A214 group | 12.38 | 2.87 | Day08-14-Day15-24 |
| OTU_364 Bacteria;Firmicutes;Clostridia;Clostridiales;Ruminococcaceae;Candidatus Soleaferrea | 8.15 | 2.80 | Day08-14-Day15-24 |
| OTU_309 Bacteria;Firmicutes;Clostridia;Clostridiales;Peptococcaceae | 6.77 | 2.57 | Day08-14-Day15-24 |
| OTU_343 Bacteria;Firmicutes;Clostridia;Clostridiales;Ruminococcaceae;Ruminiclostridium 9 | 90.41 | 2.58 | Day08-14-Day15-24 |
| OTU_16 Bacteria;Firmicutes;Clostridia;Clostridiales;Lachnospiraceae;Eisenbergiella | 683.60 | 3.70 | Day08-14-Day15-24 |
| OTU_500 Bacteria;Firmicutes;Clostridia;Clostridiales;Ruminococcaceae;Ruminococcaceae UCG-010 | 4.57 | 2.71 | Day08-14-Day15-24 |
| OTU_79 Bacteria;Firmicutes;Clostridia;Clostridiales;Clostridiales vadinBB60 group | 180.56 | 4.01 | Day08-14-Day15-24 |
| OTU_880 Bacteria;Firmicutes;Clostridia;Clostridiales;Ruminococcaceae | 7.07 | 2.97 | Day08-14-Day15-24 |
| OTU_174 Bacteria;Firmicutes;Erysipelotrichia;Erysipelotrichales;Erysipelotrichaceae;Erysipelatoclostridium | 46.11 | 2.87 | Day08-14-Day15-24 |
| OTU_6638 Bacteria;Firmicutes;Clostridia;Clostridiales;Ruminococcaceae;[Eubacterium] coprostanoligenes group | 5.68 | 2.93 | Day08-14-Day15-24 |
| OTU_264 Bacteria;Firmicutes;Clostridia;Clostridiales;Ruminococcaceae;Ruminiclostridium 5 | 8.25 | 2.88 | Day08-14-Day15-24 |
| OTU_2701 Bacteria;Firmicutes;Clostridia;Clostridiales;Lachnospiraceae;Eisenbergiella | 45.88 | 3.77 | Day08-14-Day15-24 |
| OTU_746 Bacteria;Firmicutes;Clostridia;Clostridiales;Lachnospiraceae;Lachnospiraceae UCG-010 | 6.32 | 2.88 | Day08-14-Day15-24 |
| OTU_202 Bacteria;Firmicutes;Clostridia;Clostridiales;Ruminococcaceae;Ruminiclostridium 9 | 35.96 | 2.28 | Day08-14-Day15-24 |
| OTU_646 Bacteria;Firmicutes;Clostridia;Clostridiales;Ruminococcaceae;Ruminococcaceae UCG-014 | 8.52 | 2.51 | Day08-14-Day15-24 |
| OTU_767 Bacteria;Firmicutes;Clostridia;Clostridiales;Ruminococcaceae;Ruminococcaceae UCG-014 | 9.86 | 2.54 | Day08-14-Day15-24 |
| OTU_172 Bacteria;Firmicutes;Clostridia;Clostridiales;Ruminococcaceae;Ruminiclostridium 5 | 222.20 | 2.98 | Day08-14-Day15-24 |
| OTU_424 Bacteria;Firmicutes;Clostridia;Clostridiales;Ruminococcaceae;Anaerotruncus | 12.02 | 2.38 | Day08-14-Day15-24 |
| OTU_92 Bacteria;Firmicutes;Clostridia;Clostridiales;Clostridiales vadinBB60 group | 137.86 | 2.88 | Day08-14-Day15-24 |
| OTU_564 Bacteria;Firmicutes;Clostridia;Clostridiales;Ruminococcaceae;Ruminococcaceae UCG-014 | 4.01 | 2.39 | Day08-14-Day15-24 |
| OTU_185 Bacteria;Firmicutes;Clostridia;Clostridiales;Ruminococcaceae;Ruminiclostridium 5 | 33.33 | 2.63 | Day08-14-Day15-24 |
| OTU_1277 Bacteria;Firmicutes;Clostridia;Clostridiales;Lachnospiraceae;Lachnoclostridium | 7.89 | -2.46 | Day08-14-Day15-24 |
| OTU_12336 Bacteria;Firmicutes;Clostridia;Clostridiales;Lachnospiraceae | 4.75 | 2.39 | Day08-14-Day15-24 |
| OTU_3027 Bacteria;Firmicutes;Clostridia;Clostridiales;Ruminococcaceae;Intestinimonas | 479.66 | 3.46 | Day08-14-Day15-24 |
| OTU_219 Bacteria;Firmicutes;Clostridia;Clostridiales;Lachnospiraceae | 15.89 | 3.21 | Day08-14-Day15-24 |
| OTU_67 Bacteria;Firmicutes;Clostridia;Clostridiales;Ruminococcaceae;Ruminococcaceae NK4A214 group | 209.10 | 2.56 | Day08-14-Day15-24 |
| OTU_5864 Bacteria;Firmicutes;Clostridia;Clostridiales;Ruminococcaceae;Ruminococcaceae UCG-014 | 189.15 | 3.27 | Day08-14-Day15-24 |
| OTU_217 Bacteria;Firmicutes;Clostridia;Clostridiales;Family XIII;Family XIII UCG-001 | 17.87 | 2.19 | Day08-14-Day15-24 |
| OTU_9820 Bacteria;Firmicutes;Clostridia;Clostridiales;Ruminococcaceae;Ruminiclostridium | 57.03 | 3.14 | Day08-14-Day15-24 |
| OTU_421 Bacteria;Firmicutes;Clostridia;Clostridiales;Clostridiales vadinBB60 group | 7.36 | 2.58 | Day08-14-Day15-24 |
| OTU_305 Bacteria;Firmicutes;Clostridia;Clostridiales;Ruminococcaceae;Ruminococcaceae UCG-014 | 72.72 | 2.58 | Day08-14-Day15-24 |
| OTU_17 Bacteria;Firmicutes;Clostridia;Clostridiales;Clostridiales vadinBB60 group | 165.76 | 3.90 | Day08-14-Day15-24 |
| OTU_194 Bacteria;Firmicutes;Clostridia;Clostridiales;Ruminococcaceae;Ruminococcaceae UCG-014 | 20.71 | 3.12 | Day08-14-Day15-24 |
| OTU_255 Bacteria;Firmicutes;Clostridia;Clostridiales;Ruminococcaceae;Ruminiclostridium 5 | 9.58 | 2.65 | Day08-14-Day15-24 |
| OTU_13320 Bacteria;Actinobacteria;Actinobacteria;Bifidobacteriales;Bifidobacteriaceae;Bifidobacterium;Bifidobacterium saeculare | 4.16 | 2.39 | Day08-14-Day15-24 |
| OTU_763 Bacteria;Firmicutes;Clostridia;Clostridiales;Ruminococcaceae;Ruminococcaceae UCG-005 | 10.16 | 2.37 | Day08-14-Day15-24 |
| OTU_146 Bacteria;Firmicutes;Clostridia;Clostridiales;Ruminococcaceae | 4.25 | 2.65 | Day08-14-Day15-24 |
| OTU_31 Bacteria;Firmicutes;Clostridia;Clostridiales;Ruminococcaceae;Ruminococcaceae UCG-005 | 1100.28 | 4.04 | Day08-14-Day15-24 |
| OTU_378 Bacteria;Firmicutes;Clostridia;Clostridiales;Ruminococcaceae;Ruminococcaceae UCG-014 | 4.19 | 2.51 | Day08-14-Day15-24 |
| OTU_7809 Bacteria;Firmicutes;Clostridia;Clostridiales;Lachnospiraceae;[Eubacterium] oxidoreducens group | 6.94 | 2.84 | Day08-14-Day15-24 |
| OTU_316 Bacteria;Firmicutes;Clostridia;Clostridiales;Ruminococcaceae;Ruminococcaceae UCG-013 | 4.43 | 2.64 | Day08-14-Day15-24 |
| OTU_15166 Bacteria;Firmicutes;Clostridia;Clostridiales;Ruminococcaceae;Intestinimonas | 5.87 | 2.41 | Day08-14-Day15-24 |
| OTU_317 Bacteria;Firmicutes;Clostridia;Clostridiales;Ruminococcaceae | 4.68 | 2.58 | Day08-14-Day15-24 |
| OTU_273 Bacteria;Tenericutes;Mollicutes;Mollicutes RF9 | 30.80 | 2.69 | Day08-14-Day15-24 |
| OTU_750 Bacteria;Firmicutes;Clostridia;Clostridiales;Ruminococcaceae;Ruminococcaceae NK4A214 group | 8.33 | 2.45 | Day08-14-Day15-24 |
| OTU_6846 Bacteria;Firmicutes;Clostridia;Clostridiales;Ruminococcaceae;Ruminococcaceae UCG-014 | 7.33 | 2.43 | Day08-14-Day15-24 |
| OTU_356 Bacteria;Firmicutes;Erysipelotrichia;Erysipelotrichales;Erysipelotrichaceae;Faecalitalea | 6.25 | 2.43 | Day08-14-Day15-24 |
| OTU_479 No blast hit | 3.23 | -2.01 | Day08-14-Day15-24 |
| OTU_182 Bacteria;Firmicutes;Clostridia;Clostridiales;Ruminococcaceae;[Eubacterium] coprostanoligenes group | 20.17 | 2.98 | Day08-14-Day15-24 |
| OTU_836 Bacteria;Firmicutes;Clostridia;Clostridiales;Ruminococcaceae;Ruminococcaceae UCG-014 | 5.15 | 2.14 | Day08-14-Day15-24 |
| OTU_107 Bacteria;Firmicutes;Clostridia;Clostridiales;Ruminococcaceae;Ruminiclostridium | 148.00 | 3.17 | Day08-14-Day15-24 |
| OTU_577 Bacteria;Firmicutes;Clostridia;Clostridiales;Ruminococcaceae | 3.81 | 2.38 | Day08-14-Day15-24 |
| OTU_166 Bacteria;Firmicutes;Erysipelotrichia;Erysipelotrichales;Erysipelotrichaceae;Erysipelatoclostridium | 65.59 | 2.06 | Day08-14-Day15-24 |
| OTU_6332 Bacteria;Firmicutes;Clostridia;Clostridiales;Ruminococcaceae;Flavonifractor | 3.83 | -2.15 | Day08-14-Day15-24 |
| OTU_161 Bacteria;Firmicutes;Clostridia;Clostridiales;Family XII;Fusibacter | 3.99 | 2.55 | Day08-14-Day15-24 |
| OTU_177 Bacteria;Firmicutes;Clostridia;Clostridiales;Ruminococcaceae;Ruminococcaceae UCG-009 | 24.17 | 2.97 | Day08-14-Day15-24 |
| OTU_165 Bacteria;Firmicutes;Clostridia;Clostridiales;Ruminococcaceae;Ruminococcaceae UCG-014 | 4.10 | 2.56 | Day08-14-Day15-24 |
| OTU_315 Bacteria;Firmicutes;Clostridia;Clostridiales;Lachnospiraceae | 7.89 | -2.18 | Day08-14-Day15-24 |
| OTU_210 Bacteria;Firmicutes;Clostridia;Clostridiales;Lachnospiraceae | 23.28 | 2.21 | Day08-14-Day15-24 |
| OTU_497 Bacteria;Tenericutes;Mollicutes;Mollicutes RF9 | 4.96 | 2.09 | Day08-14-Day15-24 |
| OTU_106 Bacteria;Firmicutes;Clostridia;Clostridiales;Ruminococcaceae;Ruminiclostridium 5 | 104.58 | 2.08 | Day08-14-Day15-24 |
| OTU_423 Bacteria;Firmicutes;Clostridia;Clostridiales;Lachnospiraceae;Lachnospiraceae UCG-008 | 7.32 | 2.08 | Day08-14-Day15-24 |
| OTU_13602 Bacteria;Firmicutes;Clostridia;Clostridiales;Clostridiales vadinBB60 group | 41.62 | 3.06 | Day08-14-Day15-24 |
| OTU_6277 Bacteria;Firmicutes;Clostridia;Clostridiales;Lachnospiraceae;Pseudobutyrivibrio | 87.04 | 2.87 | Day08-14-Day15-24 |
| OTU_62 Bacteria;Firmicutes;Clostridia;Clostridiales;Lachnospiraceae;Pseudobutyrivibrio | 131.08 | 2.89 | Day08-14-Day15-24 |
| OTU_1549 Bacteria;Firmicutes;Clostridia;Clostridiales;Ruminococcaceae;Ruminococcaceae UCG-014 | 3.94 | 2.10 | Day08-14-Day15-24 |
| OTU_192 Bacteria;Firmicutes;Clostridia;Clostridiales;Clostridiales vadinBB60 group | 3.99 | 2.55 | Day08-14-Day15-24 |
| OTU_508 Bacteria;Firmicutes;Clostridia;Clostridiales;Ruminococcaceae;Ruminococcaceae UCG-014 | 49.63 | 2.70 | Day08-14-Day15-24 |
| OTU_203 Bacteria;Firmicutes;Clostridia;Clostridiales;Ruminococcaceae;Ruminococcaceae UCG-002 | 11.94 | 2.47 | Day08-14-Day15-24 |
| OTU_353 Bacteria;Firmicutes;Clostridia;Clostridiales;Christensenellaceae;Christensenellaceae R-7 group | 3.95 | 2.35 | Day08-14-Day15-24 |
| OTU_71 Bacteria;Actinobacteria;Coriobacteriia;Coriobacteriales;Coriobacteriaceae;Olsenella | 12.89 | 2.88 | Day08-14-Day15-24 |
| OTU_422 Bacteria;Firmicutes;Clostridia;Clostridiales;Christensenellaceae;Christensenellaceae R-7 group | 5.90 | 2.05 | Day08-14-Day15-24 |
| OTU_367 Bacteria;Tenericutes;Mollicutes;Mollicutes RF9 | 3.25 | 2.21 | Day08-14-Day15-24 |
| OTU_291 Bacteria;Firmicutes;Clostridia;Clostridiales;Lachnospiraceae;Lachnospiraceae NK4A136 group | 3.47 | 2.32 | Day08-14-Day15-24 |
| OTU_68 Bacteria;Firmicutes;Clostridia;Clostridiales;Ruminococcaceae;Hydrogenoanaerobacterium | 99.81 | 3.08 | Day08-14-Day15-24 |
| OTU_1194 Bacteria;Firmicutes;Clostridia;Clostridiales;Ruminococcaceae;Ruminococcaceae UCG-014 | 14.01 | 2.35 | Day08-14-Day15-24 |
| OTU_132 Bacteria;Firmicutes;Clostridia;Clostridiales;Ruminococcaceae;Ruminiclostridium 5 | 90.58 | 2.35 | Day08-14-Day15-24 |
| OTU_282 Bacteria;Firmicutes;Clostridia;Clostridiales;Ruminococcaceae;Ruminococcaceae UCG-005 | 3.15 | 2.16 | Day08-14-Day15-24 |
| OTU_311 Bacteria;Firmicutes;Clostridia;Clostridiales;Clostridiales vadinBB60 group | 3.33 | 2.25 | Day08-14-Day15-24 |
| OTU_285 Bacteria;Firmicutes;Clostridia;Clostridiales;Lachnospiraceae;Roseburia | 15.67 | 2.03 | Day08-14-Day15-24 |
| OTU_322 Bacteria;Firmicutes;Clostridia;Clostridiales;Lachnospiraceae;Tyzzerella | 3.16 | 2.19 | Day08-14-Day15-24 |
| OTU_390 Bacteria;Firmicutes;Clostridia;Clostridiales;Ruminococcaceae;Ruminococcaceae UCG-014 | 9.20 | 2.18 | Day08-14-Day15-24 |
| OTU_605 Bacteria;Firmicutes;Clostridia;Clostridiales;Ruminococcaceae;Ruminococcaceae UCG-014 | 63.31 | 2.07 | Day08-14-Day15-24 |
| OTU_3471 Bacteria;Actinobacteria;Coriobacteriia;Coriobacteriales;Coriobacteriaceae;Olsenella | 7.95 | 2.54 | Day08-14-Day15-24 |
| OTU_47 Bacteria;Firmicutes;Clostridia;Clostridiales;Clostridiales vadinBB60 group | 53.47 | 2.70 | Day08-14-Day15-24 |
| OTU_2618 Bacteria;Firmicutes;Clostridia;Clostridiales;Ruminococcaceae;Intestinimonas | 49.41 | 2.38 | Day08-14-Day15-24 |
| OTU_168 Bacteria;Firmicutes;Clostridia;Clostridiales;Ruminococcaceae;Ruminococcaceae UCG-014 | 226.03 | 2.17 | Day08-14-Day15-24 |
| OTU_296 Bacteria;Firmicutes;Clostridia;Clostridiales;Peptostreptococcaceae;Intestinibacter | 5.42 | 2.08 | Day08-14-Day15-24 |
| OTU_198 Bacteria;Firmicutes;Clostridia;Clostridiales;Ruminococcaceae;Faecalibacterium | 43.74 | 2.23 | Day08-14-Day15-24 |
| OTU_4604 Bacteria;Firmicutes;Clostridia;Clostridiales;Clostridiales vadinBB60 group | 12.66 | 2.43 | Day08-14-Day15-24 |
| OTU_89 Bacteria;Firmicutes;Clostridia;Clostridiales;Ruminococcaceae;Ruminococcaceae UCG-014 | 119.98 | 2.03 | Day08-14-Day15-24 |
| OTU_2398 Bacteria;Firmicutes;Clostridia;Clostridiales;Ruminococcaceae | 3.54 | 2.00 | Day08-14-Day15-24 |
| OTU_6012 Bacteria;Firmicutes;Clostridia;Clostridiales;Ruminococcaceae;Hydrogenoanaerobacterium | 55.27 | 2.67 | Day08-14-Day15-24 |
| OTU_64 Bacteria;Firmicutes;Clostridia;Clostridiales;Ruminococcaceae;Ruminiclostridium 5 | 57.47 | 2.89 | Day08-14-Day15-24 |
| OTU_261 Bacteria;Firmicutes;Clostridia;Clostridiales;Christensenellaceae;Christensenellaceae R-7 group | 2.93 | 2.04 | Day08-14-Day15-24 |
| OTU_271 Bacteria;Firmicutes;Clostridia;Clostridiales;Lachnospiraceae | 5.35 | 2.09 | Day08-14-Day15-24 |
| OTU_12293 Bacteria;Firmicutes;Clostridia;Clostridiales;Ruminococcaceae;Ruminiclostridium 5 | 44.09 | 2.22 | Day08-14-Day15-24 |
| OTU_178 Bacteria;Firmicutes;Clostridia;Clostridiales;Ruminococcaceae;Anaerotruncus | 53.06 | 2.09 | Day08-14-Day15-24 |
| OTU_109 Bacteria;Firmicutes;Clostridia;Clostridiales;Ruminococcaceae;Ruminococcaceae UCG-014 | 171.14 | 2.06 | Day08-14-Day15-24 |
| OTU_6707 Bacteria;Firmicutes;Clostridia;Clostridiales;Ruminococcaceae | 196.56 | 2.59 | Day08-14-Day15-24 |
| OTU_3463 Bacteria;Firmicutes;Clostridia;Clostridiales;Ruminococcaceae | 232.87 | 2.24 | Day08-14-Day15-24 |
| OTU_21 Bacteria;Firmicutes;Clostridia;Clostridiales;Ruminococcaceae | 511.16 | 2.44 | Day08-14-Day15-24 |
| OTU_218 Bacteria;Firmicutes;Clostridia;Clostridiales;Ruminococcaceae;Butyricicoccus;Butyricicoccus pullicaecorum 1.2 | 50.48 | 2.03 | Day08-14-Day15-24 |
| OTU_293 Bacteria;Firmicutes;Clostridia;Clostridiales;Clostridiales vadinBB60 group | 102.58 | 2.38 | Day08-14-Day15-24 |
| OTU_145 Bacteria;Firmicutes;Clostridia;Clostridiales;Clostridiales vadinBB60 group | 139.30 | 7.98 | Day15-24-Day25-35 |
| OTU_58 Bacteria;Cyanobacteria;Melainabacteria;Gastranaerophilales | 900.13 | 9.81 | Day15-24-Day25-35 |
| OTU_160 Bacteria;Firmicutes;Clostridia;Thermoanaerobacterales;Thermoanaerobacteraceae;Gelria | 82.01 | 6.61 | Day15-24-Day25-35 |
| OTU_143 Bacteria;Firmicutes;Clostridia;Clostridiales;Clostridiales vadinBB60 group | 120.12 | 7.06 | Day15-24-Day25-35 |
| OTU_221 Bacteria;Firmicutes;Clostridia;Clostridiales;Lachnospiraceae;Lachnoclostridium | 48.19 | 6.25 | Day15-24-Day25-35 |
| OTU_85 Bacteria;Firmicutes;Clostridia;Clostridiales;Clostridiales vadinBB60 group | 73.47 | 7.07 | Day15-24-Day25-35 |
| OTU_288 Bacteria;Firmicutes;Clostridia;Clostridiales;Ruminococcaceae;Anaerotruncus | 28.95 | 4.91 | Day15-24-Day25-35 |
| OTU_120 Bacteria;Firmicutes;Clostridia;Clostridiales;Clostridiales vadinBB60 group | 97.46 | 6.93 | Day15-24-Day25-35 |
| OTU_211 Bacteria;Tenericutes;Mollicutes;NB1-n | 55.87 | 6.57 | Day15-24-Day25-35 |
| OTU_308 Bacteria;Firmicutes;Clostridia;Clostridiales;Ruminococcaceae;Ruminococcaceae UCG-014 | 21.44 | 5.28 | Day15-24-Day25-35 |
| OTU_343 Bacteria;Firmicutes;Clostridia;Clostridiales;Ruminococcaceae;Ruminiclostridium 9 | 620.73 | 2.98 | Day15-24-Day25-35 |
| OTU_341 Bacteria;Firmicutes;Clostridia;Clostridiales;Ruminococcaceae;Ruminococcaceae UCG-014 | 17.13 | 4.53 | Day15-24-Day25-35 |
| OTU_625 Bacteria;Firmicutes;Clostridia;Clostridiales;Ruminococcaceae | 29.50 | 4.00 | Day15-24-Day25-35 |
| OTU_13431 Bacteria;Firmicutes;Clostridia;Thermoanaerobacterales;Thermoanaerobacteraceae;Gelria | 34.18 | 5.11 | Day15-24-Day25-35 |
| OTU_8121 Bacteria;Firmicutes;Clostridia;Clostridiales;Clostridiales vadinBB60 group | 31.11 | 5.85 | Day15-24-Day25-35 |
| OTU_430 Bacteria;Firmicutes;Clostridia;Clostridiales;Lachnospiraceae | 9.34 | 3.40 | Day15-24-Day25-35 |
| OTU_347 Bacteria;Firmicutes;Clostridia;Clostridiales;Ruminococcaceae;Ruminococcaceae UCG-010 | 18.21 | 5.03 | Day15-24-Day25-35 |
| OTU_146 Bacteria;Firmicutes;Clostridia;Clostridiales;Ruminococcaceae | 109.29 | 4.96 | Day15-24-Day25-35 |
| OTU_293 Bacteria;Firmicutes;Clostridia;Clostridiales;Clostridiales vadinBB60 group | 98.26 | -5.68 | Day15-24-Day25-35 |
| OTU_339 Bacteria;Firmicutes;Clostridia;Clostridiales;Ruminococcaceae;Ruminiclostridium 5 | 17.88 | 2.86 | Day15-24-Day25-35 |
| OTU_350 Bacteria;Firmicutes;Clostridia;Clostridiales;Ruminococcaceae | 17.76 | 3.21 | Day15-24-Day25-35 |
| OTU_496 Bacteria;Firmicutes;Clostridia;Clostridiales;Ruminococcaceae | 8.16 | 2.89 | Day15-24-Day25-35 |
| OTU_671 Bacteria;Firmicutes;Clostridia;Clostridiales;Ruminococcaceae;Ruminococcaceae UCG-014 | 13.18 | 4.04 | Day15-24-Day25-35 |
| OTU_144 Bacteria;Firmicutes;Clostridia;Clostridiales;Clostridiales vadinBB60 group | 51.35 | -4.37 | Day15-24-Day25-35 |
| OTU_338 Bacteria;Firmicutes;Clostridia;Clostridiales;Ruminococcaceae | 18.09 | 3.71 | Day15-24-Day25-35 |
| OTU_298 Bacteria;Firmicutes;Clostridia;Clostridiales;Ruminococcaceae;Ruminiclostridium 9 | 116.07 | 2.55 | Day15-24-Day25-35 |
| OTU_244 Bacteria;Firmicutes;Clostridia;Clostridiales;Clostridiales vadinBB60 group | 29.45 | 5.01 | Day15-24-Day25-35 |
| OTU_404 Bacteria;Firmicutes;Clostridia;Clostridiales;Ruminococcaceae;Ruminococcaceae UCG-002 | 10.09 | 3.86 | Day15-24-Day25-35 |
| OTU_640 Bacteria;Firmicutes;Clostridia;Clostridiales;Clostridiales vadinBB60 group | 12.65 | 4.39 | Day15-24-Day25-35 |
| OTU_1020 Bacteria;Firmicutes;Clostridia;Clostridiales;Ruminococcaceae;Ruminococcaceae UCG-014 | 23.11 | 4.15 | Day15-24-Day25-35 |
| OTU_277 Bacteria;Firmicutes;Clostridia;Clostridiales;Clostridiales vadinBB60 group | 24.63 | 4.62 | Day15-24-Day25-35 |
| OTU_697 Bacteria;Firmicutes;Clostridia;Clostridiales;Ruminococcaceae;Ruminococcaceae UCG-014 | 11.56 | 4.11 | Day15-24-Day25-35 |
| OTU_216 Bacteria;Firmicutes;Clostridia;Clostridiales;Clostridiales vadinBB60 group | 46.16 | 4.37 | Day15-24-Day25-35 |
| OTU_282 Bacteria;Firmicutes;Clostridia;Clostridiales;Ruminococcaceae;Ruminococcaceae UCG-005 | 31.78 | 3.32 | Day15-24-Day25-35 |
| OTU_161 Bacteria;Firmicutes;Clostridia;Clostridiales;Family XII;Fusibacter | 132.46 | 4.35 | Day15-24-Day25-35 |
| OTU_355 Bacteria;Firmicutes;Clostridia;Clostridiales;Clostridiales vadinBB60 group | 12.62 | 4.27 | Day15-24-Day25-35 |
| OTU_410 Bacteria;Firmicutes;Clostridia;Clostridiales;Ruminococcaceae;Subdoligranulum | 23.11 | 4.51 | Day15-24-Day25-35 |
| OTU_379 Bacteria;Firmicutes;Clostridia;Clostridiales;Ruminococcaceae;[Eubacterium] coprostanoligenes group | 7.50 | 3.68 | Day15-24-Day25-35 |
| OTU_530 Bacteria;Actinobacteria;Coriobacteriia;Coriobacteriales;Coriobacteriaceae;Gordonibacter | 4.96 | 2.88 | Day15-24-Day25-35 |
| OTU_141 Bacteria;Firmicutes;Clostridia;Clostridiales;Lachnospiraceae | 194.57 | 4.91 | Day15-24-Day25-35 |
| OTU_542 Bacteria;Firmicutes;Clostridia;Clostridiales;Clostridiaceae 1 | 6.06 | 2.87 | Day15-24-Day25-35 |
| OTU_572 Bacteria;Firmicutes;Clostridia;Clostridiales;Ruminococcaceae | 5.30 | 3.18 | Day15-24-Day25-35 |
| OTU_577 Bacteria;Firmicutes;Clostridia;Clostridiales;Ruminococcaceae | 28.51 | 3.14 | Day15-24-Day25-35 |
| OTU_280 Bacteria;Firmicutes;Clostridia;Clostridiales;Ruminococcaceae;Anaerotruncus | 21.26 | 3.22 | Day15-24-Day25-35 |
| OTU_6791 Bacteria;Firmicutes;Clostridia;Clostridiales;Ruminococcaceae;Ruminococcaceae UCG-004 | 42.99 | 2.38 | Day15-24-Day25-35 |
| OTU_2200 Bacteria;Firmicutes;Clostridia;Clostridiales;Ruminococcaceae;Ruminococcaceae UCG-014 | 7.14 | 3.14 | Day15-24-Day25-35 |
| OTU_278 Bacteria;Firmicutes;Clostridia;Clostridiales;Ruminococcaceae;Anaerotruncus | 16.49 | -3.13 | Day15-24-Day25-35 |
| OTU_115 Bacteria;Actinobacteria;Coriobacteriia;Coriobacteriales;Coriobacteriaceae;Collinsella;Enorma massiliensis phI | 184.33 | 4.87 | Day15-24-Day25-35 |
| OTU_557 Bacteria;Tenericutes;Mollicutes;Mollicutes RF9 | 5.43 | 2.82 | Day15-24-Day25-35 |
| OTU_270 Bacteria;Firmicutes;Clostridia;Clostridiales;Ruminococcaceae;Anaerotruncus | 31.95 | 2.62 | Day15-24-Day25-35 |
| OTU_165 Bacteria;Firmicutes;Clostridia;Clostridiales;Ruminococcaceae;Ruminococcaceae UCG-014 | 149.80 | 4.40 | Day15-24-Day25-35 |
| OTU_549 Bacteria;Firmicutes;Clostridia;Clostridiales;Peptococcaceae | 5.28 | 3.17 | Day15-24-Day25-35 |
| OTU_481 Bacteria;Firmicutes;Clostridia;Clostridiales;Ruminococcaceae;Hydrogenoanaerobacterium | 6.78 | 2.29 | Day15-24-Day25-35 |
| OTU_398 Bacteria;Firmicutes;Clostridia;Clostridiales;Lachnospiraceae;Roseburia | 6.82 | 3.54 | Day15-24-Day25-35 |
| OTU_392 Bacteria;Firmicutes;Clostridia;Clostridiales;Ruminococcaceae;Ruminiclostridium 9 | 14.64 | 2.21 | Day15-24-Day25-35 |
| OTU_3813 Bacteria;Firmicutes;Clostridia;Clostridiales;Ruminococcaceae;Anaerotruncus | 10.60 | -2.95 | Day15-24-Day25-35 |
| OTU_400 Bacteria;Firmicutes;Clostridia;Clostridiales;Ruminococcaceae;Anaerotruncus | 10.69 | 2.81 | Day15-24-Day25-35 |
| OTU_322 Bacteria;Firmicutes;Clostridia;Clostridiales;Lachnospiraceae;Tyzzerella | 21.49 | 3.01 | Day15-24-Day25-35 |
| OTU_140 Bacteria;Firmicutes;Clostridia;Clostridiales;Ruminococcaceae;Ruminococcaceae UCG-004 | 107.30 | 2.43 | Day15-24-Day25-35 |
| OTU_365 Bacteria;Firmicutes;Clostridia;Clostridiales;Ruminococcaceae | 9.73 | 3.25 | Day15-24-Day25-35 |
| OTU_763 Bacteria;Firmicutes;Clostridia;Clostridiales;Ruminococcaceae;Ruminococcaceae UCG-005 | 47.88 | 2.38 | Day15-24-Day25-35 |
| OTU_520 Bacteria;Firmicutes;Clostridia;Clostridiales;Peptococcaceae | 6.91 | 2.85 | Day15-24-Day25-35 |
| OTU_276 Bacteria;Proteobacteria;Gammaproteobacteria;Pasteurellales;Pasteurellaceae;Gallibacterium;Gallibacterium anatis | 14.63 | 3.35 | Day15-24-Day25-35 |
| OTU_47 Bacteria;Firmicutes;Clostridia;Clostridiales;Clostridiales vadinBB60 group | 531.12 | 3.55 | Day15-24-Day25-35 |
| OTU_585 Bacteria;Firmicutes;Clostridia;Clostridiales;Ruminococcaceae;Ruminococcaceae UCG-010 | 3.94 | 2.29 | Day15-24-Day25-35 |
| OTU_311 Bacteria;Firmicutes;Clostridia;Clostridiales;Clostridiales vadinBB60 group | 55.18 | 3.47 | Day15-24-Day25-35 |
| OTU_580 Bacteria;Tenericutes;Mollicutes;Mollicutes RF9 | 3.86 | 2.55 | Day15-24-Day25-35 |
| OTU_3471 Bacteria;Actinobacteria;Coriobacteriia;Coriobacteriales;Coriobacteriaceae;Olsenella | 197.09 | 4.30 | Day15-24-Day25-35 |
| OTU_548 Bacteria;Firmicutes;Clostridia;Clostridiales;Peptococcaceae | 6.75 | 2.33 | Day15-24-Day25-35 |
| OTU_2760 Bacteria;Firmicutes;Clostridia;Clostridiales;Ruminococcaceae;Anaerotruncus | 189.84 | -2.95 | Day15-24-Day25-35 |
| OTU_46 Bacteria;Firmicutes;Clostridia;Clostridiales;Ruminococcaceae;Anaerotruncus | 359.69 | -2.94 | Day15-24-Day25-35 |
| OTU_200 Bacteria;Firmicutes;Clostridia;Clostridiales;Clostridiales vadinBB60 group | 6.22 | 3.34 | Day15-24-Day25-35 |
| OTU_302 Bacteria;Firmicutes;Clostridia;Clostridiales;Lachnospiraceae;Lachnospiraceae FCS020 group | 21.20 | -2.37 | Day15-24-Day25-35 |
| OTU_304 Bacteria;Firmicutes;Clostridia;Clostridiales;Ruminococcaceae;Ruminococcaceae UCG-010 | 25.57 | 2.90 | Day15-24-Day25-35 |
| OTU_294 Bacteria;Cyanobacteria;Melainabacteria;Gastranaerophilales | 5.32 | 3.14 | Day15-24-Day25-35 |
| OTU_71 Bacteria;Actinobacteria;Coriobacteriia;Coriobacteriales;Coriobacteriaceae;Olsenella | 321.67 | 4.29 | Day15-24-Day25-35 |
| OTU_203 Bacteria;Firmicutes;Clostridia;Clostridiales;Ruminococcaceae;Ruminococcaceae UCG-002 | 56.27 | 2.37 | Day15-24-Day25-35 |
| OTU_187 Bacteria;Firmicutes;Clostridia;Clostridiales;Ruminococcaceae;Ruminococcaceae UCG-014 | 32.88 | 3.78 | Day15-24-Day25-35 |
| OTU_547 Bacteria;Firmicutes;Clostridia;Clostridiales;Ruminococcaceae;Intestinimonas | 6.03 | 2.32 | Day15-24-Day25-35 |
| OTU_15166 Bacteria;Firmicutes;Clostridia;Clostridiales;Ruminococcaceae;Intestinimonas | 33.90 | 2.72 | Day15-24-Day25-35 |
| OTU_425 Bacteria;Firmicutes;Clostridia;Clostridiales;Clostridiales vadinBB60 group | 4.72 | 2.80 | Day15-24-Day25-35 |
| OTU_442 Bacteria;Firmicutes;Clostridia;Clostridiales;Ruminococcaceae;Ruminococcaceae UCG-013 | 9.14 | 2.42 | Day15-24-Day25-35 |
| OTU_281 Bacteria;Firmicutes;Clostridia;Clostridiales;Ruminococcaceae;Ruminococcaceae UCG-014 | 27.93 | 2.94 | Day15-24-Day25-35 |
| OTU_158 Bacteria;Firmicutes;Clostridia;Clostridiales;Ruminococcaceae;Ruminococcaceae UCG-008 | 37.33 | 3.20 | Day15-24-Day25-35 |
| OTU_579 Bacteria;Firmicutes;Clostridia;Clostridiales;Ruminococcaceae;Ruminococcaceae NK4A214 group | 5.14 | 2.28 | Day15-24-Day25-35 |
| OTU_13511 Bacteria;Firmicutes;Clostridia;Clostridiales;Ruminococcaceae;Ruminococcaceae UCG-005 | 5.65 | 2.37 | Day15-24-Day25-35 |
| OTU_372 Bacteria;Actinobacteria;Coriobacteriia;Coriobacteriales;Coriobacteriaceae;Coriobacteriaceae UCG-002 | 8.28 | 2.73 | Day15-24-Day25-35 |
| OTU_250 Bacteria;Firmicutes;Clostridia;Clostridiales;Ruminococcaceae | 37.27 | 2.25 | Day15-24-Day25-35 |
| OTU_76 Bacteria;Firmicutes;Clostridia;Clostridiales;Ruminococcaceae;Ruminococcaceae UCG-014 | 415.79 | 3.79 | Day15-24-Day25-35 |
| OTU_630 Bacteria;Firmicutes;Clostridia;Clostridiales;Ruminococcaceae;Intestinimonas | 106.71 | 2.89 | Day15-24-Day25-35 |
| OTU_853 Bacteria;Firmicutes;Clostridia;Clostridiales;Peptococcaceae | 2.85 | 2.14 | Day15-24-Day25-35 |
| OTU_128 Bacteria;Firmicutes;Clostridia;Clostridiales;Ruminococcaceae;[Eubacterium] coprostanoligenes group | 80.82 | -3.70 | Day15-24-Day25-35 |
| OTU_332 Bacteria;Firmicutes;Clostridia;Clostridiales;Ruminococcaceae;Ruminococcaceae UCG-008 | 5.84 | 2.83 | Day15-24-Day25-35 |
| OTU_153 Bacteria;Firmicutes;Clostridia;Clostridiales;Lachnospiraceae;[Eubacterium] oxidoreducens group | 101.01 | 2.81 | Day15-24-Day25-35 |
| OTU_13399 Bacteria;Firmicutes;Clostridia;Clostridiales;Ruminococcaceae;Ruminococcaceae UCG-002 | 97.82 | 2.49 | Day15-24-Day25-35 |
| OTU_680 Bacteria;Firmicutes;Clostridia;Clostridiales;Ruminococcaceae;Ruminococcaceae UCG-014 | 9.12 | 2.91 | Day15-24-Day25-35 |
| OTU_7809 Bacteria;Firmicutes;Clostridia;Clostridiales;Lachnospiraceae;[Eubacterium] oxidoreducens group | 54.13 | 2.64 | Day15-24-Day25-35 |
| OTU_1505 Bacteria;Firmicutes;Clostridia;Clostridiales;Ruminococcaceae;Ruminococcaceae UCG-014 | 3.65 | 2.21 | Day15-24-Day25-35 |
| OTU_317 Bacteria;Firmicutes;Clostridia;Clostridiales;Ruminococcaceae | 22.02 | 2.36 | Day15-24-Day25-35 |
| OTU_436 No blast hit | 3.45 | 2.48 | Day15-24-Day25-35 |
| OTU_464 Bacteria;Firmicutes;Clostridia;Clostridiales;Ruminococcaceae;Ruminiclostridium 5 | 3.98 | 2.52 | Day15-24-Day25-35 |
| OTU_291 Bacteria;Firmicutes;Clostridia;Clostridiales;Lachnospiraceae;Lachnospiraceae NK4A136 group | 18.43 | 2.58 | Day15-24-Day25-35 |
| OTU_6396 Bacteria;Firmicutes;Clostridia;Clostridiales;Clostridiales vadinBB60 group | 512.09 | 3.06 | Day15-24-Day25-35 |
| OTU_775 Bacteria;Firmicutes;Clostridia;Clostridiales;Peptococcaceae | 2.85 | 2.16 | Day15-24-Day25-35 |
| OTU_381 Bacteria;Firmicutes;Clostridia;Clostridiales;Clostridiales vadinBB60 group | 46.10 | 2.15 | Day15-24-Day25-35 |
| OTU_503 No blast hit | 3.74 | 2.03 | Day15-24-Day25-35 |
| OTU_135 Bacteria;Firmicutes;Clostridia;Clostridiales;Ruminococcaceae;Ruminococcaceae NK4A214 group | 260.87 | 3.08 | Day15-24-Day25-35 |
| OTU_3901 Bacteria;Proteobacteria;Gammaproteobacteria;Enterobacteriales;Enterobacteriaceae;Escherichia-Shigella;Shigella flexneri K-671 | 14.27 | 2.32 | Day15-24-Day25-35 |
| OTU_655 Bacteria;Firmicutes;Clostridia;Clostridiales;Ruminococcaceae;Ruminiclostridium | 2.77 | 2.10 | Day15-24-Day25-35 |
| OTU_380 Bacteria;Firmicutes;Clostridia;Clostridiales;Clostridiales vadinBB60 group | 422.96 | 2.15 | Day15-24-Day25-35 |
| OTU_100 Bacteria;Firmicutes;Clostridia;Clostridiales;Clostridiales vadinBB60 group | 402.31 | 2.43 | Day15-24-Day25-35 |
| OTU_2893 Bacteria;Firmicutes;Clostridia;Clostridiales;Ruminococcaceae;Ruminococcaceae UCG-014 | 4.84 | 2.08 | Day15-24-Day25-35 |
| OTU_761 Bacteria;Firmicutes;Clostridia;Clostridiales;Ruminococcaceae;Hydrogenoanaerobacterium | 2.67 | 2.04 | Day15-24-Day25-35 |
| OTU_6645 Bacteria;Firmicutes;Clostridia;Clostridiales;Clostridiales vadinBB60 group | 179.75 | 3.40 | Day15-24-Day25-35 |
| OTU_534 Bacteria;Firmicutes;Clostridia;Clostridiales;Ruminococcaceae;Ruminiclostridium 5 | 8.06 | 2.02 | Day15-24-Day25-35 |
| OTU_555 No blast hit | 3.26 | 2.05 | Day15-24-Day25-35 |
| OTU_491 Bacteria;Firmicutes;Clostridia;Clostridiales;Ruminococcaceae;Ruminococcaceae UCG-014 | 10.10 | 2.32 | Day15-24-Day25-35 |
| OTU_439 Bacteria;Lentisphaerae;Lentisphaeria;Victivallales;Victivallaceae;Victivallis;Victivallis vadensis | 2.98 | 2.18 | Day15-24-Day25-35 |
| OTU_177 Bacteria;Firmicutes;Clostridia;Clostridiales;Ruminococcaceae;Ruminococcaceae UCG-009 | 114.43 | 2.33 | Day15-24-Day25-35 |
| OTU_169 Bacteria;Firmicutes;Clostridia;Clostridiales;Lachnospiraceae | 158.72 | 2.51 | Day15-24-Day25-35 |
| OTU_252 Bacteria;Firmicutes;Clostridia;Clostridiales;Lachnospiraceae;Coprococcus 1 | 29.25 | 2.37 | Day15-24-Day25-35 |
| OTU_271 Bacteria;Firmicutes;Clostridia;Clostridiales;Lachnospiraceae | 48.59 | 2.32 | Day15-24-Day25-35 |
| OTU_245 Bacteria;Firmicutes;Clostridia;Clostridiales;Ruminococcaceae | 33.84 | 2.44 | Day15-24-Day25-35 |
| OTU_38 Bacteria;Firmicutes;Clostridia;Clostridiales;Ruminococcaceae | 529.38 | -2.14 | Day15-24-Day25-35 |
| OTU_97 Bacteria;Firmicutes;Clostridia;Clostridiales;Christensenellaceae;Christensenellaceae R-7 group | 287.01 | 2.03 | Day15-24-Day25-35 |
| OTU_1938 Bacteria;Firmicutes;Clostridia;Clostridiales;Ruminococcaceae | 956.00 | 2.61 | Day15-24-Day25-35 |
| OTU_156 Bacteria;Firmicutes;Clostridia;Clostridiales;Ruminococcaceae;Oscillospira | 133.49 | 2.03 | Day15-24-Day25-35 |
| OTU_261 Bacteria;Firmicutes;Clostridia;Clostridiales;Christensenellaceae;Christensenellaceae R-7 group | 37.88 | 2.33 | Day15-24-Day25-35 |
| OTU_316 Bacteria;Firmicutes;Clostridia;Clostridiales;Ruminococcaceae;Ruminococcaceae UCG-013 | 17.58 | 2.05 | Day15-24-Day25-35 |
| OTU_627 Bacteria;Firmicutes;Clostridia;Clostridiales;Lachnospiraceae;[Eubacterium] hallii group | 9.47 | 2.01 | Day15-24-Day25-35 |
| OTU_209 Bacteria;Firmicutes;Clostridia;Clostridiales;Clostridiales vadinBB60 group | 63.75 | 2.22 | Day15-24-Day25-35 |
| OTU_4604 Bacteria;Firmicutes;Clostridia;Clostridiales;Clostridiales vadinBB60 group | 297.46 | 2.37 | Day15-24-Day25-35 |
| OTU_5826 Bacteria;Firmicutes;Clostridia;Clostridiales;Ruminococcaceae | 191.24 | 2.25 | Day15-24-Day25-35 |
| OTU_8773 Bacteria;Firmicutes;Clostridia;Clostridiales;Ruminococcaceae;[Eubacterium] coprostanoligenes group | 264.42 | 2.38 | Day15-24-Day25-35 |
| OTU_7 Bacteria;Firmicutes;Clostridia;Clostridiales;Ruminococcaceae | 1138.83 | 2.43 | Day15-24-Day25-35 |
| OTU_201 Bacteria;Firmicutes;Clostridia;Clostridiales;Clostridiales vadinBB60 group | 93.80 | 2.33 | Day15-24-Day25-35 |
| OTU_102 Bacteria;Firmicutes;Clostridia;Clostridiales;Ruminococcaceae;Ruminiclostridium 9 | 327.51 | 2.08 | Day15-24-Day25-35 |
| OTU_42 Bacteria;Firmicutes;Clostridia;Clostridiales;Clostridiales vadinBB60 group | 503.33 | 2.42 | Day15-24-Day25-35 |
| OTU_17 Bacteria;Firmicutes;Clostridia;Clostridiales;Clostridiales vadinBB60 group | 1202.77 | 2.37 | Day15-24-Day25-35 |
| OTU_66 Bacteria;Firmicutes;Clostridia;Clostridiales;Ruminococcaceae | 367.68 | 2.23 | Day15-24-Day25-35 |
| OTU_15345 Bacteria;Firmicutes;Clostridia;Clostridiales;Clostridiales vadinBB60 group | 46.80 | 2.14 | Day15-24-Day25-35 |
| OTU_29 Bacteria;Firmicutes;Clostridia;Clostridiales;Ruminococcaceae;[Eubacterium] coprostanoligenes group | 694.79 | 2.37 | Day15-24-Day25-35 |
| OTU_15527 Bacteria;Firmicutes;Clostridia;Clostridiales;Ruminococcaceae;Ruminococcaceae UCG-014 | 114.74 | 2.02 | Day15-24-Day25-35 |
| OTU_192 Bacteria;Firmicutes;Clostridia;Clostridiales;Clostridiales vadinBB60 group | 48.17 | 2.23 | Day15-24-Day25-35 |
