# Supplementary material for: Comprehensive Longitudinal Microbiome Analysis of the Chicken Cecum Reveals a Shift From Competitive to Environmental Drivers and a Window of Opportunity for Campylobacter
Source: Front Microbiol. 2018 Oct 15;9:2452. doi: 10.3389/fmicb.2018.02452 (PMC6196313; doi:10.3389/fmicb.2018.02452)
Supplement: Supplementary Table 2 — Differential analysis of genera that are up/down regulated between different groups (Adjusted P values ≤ 0.05) where positive log2 fold change represent genera becoming abundant as we go forward in time. Here only the significant genera are shown for daily comparisons. [file Table_2.DOCX]

Supplementary Table 2: Differential analysis of genera that are up/down regulated between different groups (Adjusted P values ≤ 0.05) where positive log2 fold change represent genera becoming abundant as we go forward in time. Here only the significant genera are shown for daily comparisons.

| Genus | Base Mean | Log2 Fold | Group |
| --- | --- | --- | --- |
| Ruminococcaceae UCG-002 | 27.48 | 4.73 | 03-04 |
| Ruminococcaceae UCG-014 | 84.78 | 4.17 | 03-04 |
| Ruminococcaceae UCG-005 | 10.12 | 3.50 | 03-04 |
| Lachnospiraceae;Lachnospira | 19.72 | 2.71 | 04-05 |
| [Eubacterium] hallii group | 83.69 | 2.48 | 04-05 |
| Enterobacter | 1975.82 | -2.55 | 04-05 |
| Candidatus Arthromitus | 5.21 | 2.54 | 05-06 |
| Prevotellaceae;Prevotella 7 | 3.27 | 2.13 | 05-06 |
| Tyzzerella | 7.12 | 2.56 | 05-06 |
| Anaerofilum | 4.03 | 2.25 | 05-06 |
| Enterococcus | 65.50 | -3.33 | 05-06 |
| Defluviitaleaceae UCG-011 | 24.57 | -3.43 | 05-06 |
| Lachnospiraceae UCG-010 | 160.54 | 2.97 | 06-07 |
| Coprococcus 1 | 1124.60 | 2.09 | 06-07 |
| Shuttleworthia | 145.95 | 2.13 | 06-07 |
| Brevibacillus | 4.21 | -2.27 | 06-07 |
| Prevotella 7 | 3.12 | -2.06 | 06-07 |
| Tyzzerella | 73.83 | 2.90 | 07-08 |
| Faecalibacterium | 405.59 | 4.04 | 08-09 |
| Enterobacter | 35.00 | -3.30 | 08-09 |
| Family XIII UCG-001 | 4.18 | 2.19 | 09-10 |
| Roseburia | 10.30 | 2.26 | 09-10 |
| Tyzzerella | 488.55 | -2.38 | 09-10 |
| Corynebacterium 1 | 5.29 | 2.10 | 10-11 |
| Ruminococcaceae UCG-008 | 4.46 | 2.17 | 10-11 |
| Tyzzerella | 492.04 | 2.76 | 10-11 |
| Fusicatenibacter | 694.62 | -3.07 | 10-11 |
| Lachnospiraceae FE2018 group | 507.70 | -2.97 | 10-11 |
| Ruminococcus 1 | 19.47 | -2.54 | 10-11 |
| Defluviitaleaceae UCG-011 | 212.59 | -2.44 | 10-11 |
| Ruminococcaceae UCG-005 | 783.84 | -2.24 | 10-11 |
| Comamonadaceae; Delftia | 5.50 | 2.03 | 12-13 |
| Ruminococcus 1 | 50.97 | 3.13 | 12-13 |
| Butyricicoccus | 26.20 | 3.16 | 12-13 |
| Megamonas | 13.32 | -2.73 | 12-13 |
| Faecalitalea | 37.63 | 3.47 | 13-14 |
| Enterorhabdus | 6.48 | -2.03 | 13-14 |
| Ruminococcus 1 | 64.03 | 2.17 | 14-15 |
| Acetitomaculum | 38.98 | 2.17 | 14-15 |
| Family XIII UCG-001 | 20.64 | 2.39 | 14-15 |
| Bifidobacterium | 83.86 | 2.54 | 14-15 |
| Lachnospiraceae FCS020 group | 303.03 | 2.57 | 14-15 |
| Lactobacillus | 1058.97 | 2.80 | 14-15 |
| Butyricicoccus | 118.45 | 3.01 | 14-15 |
| Faecalitalea | 35.59 | -4.53 | 14-15 |
| Escherichia-Shigella | 3107.38 | -2.08 | 14-15 |
| Enterobacteriaceae;Enterobacter | 3.81 | -2.05 | 14-15 |
| Streptococcus | 8.08 | 2.91 | 15-16 |
| Ruminococcaceae UCG-009 | 334.48 | 2.44 | 16-17 |
| Ruminococcaceae V9D2013 group | 10.84 | 3.39 | 16-17 |
| Erysipelatoclostridium | 423.97 | -2.09 | 16-17 |
| Marvinbryantia | 22.84 | -2.70 | 16-17 |
| Intestinimonas | 538.49 | 2.23 | 16-17 |
| Tyzzerella 3 | 171.81 | -2.19 | 16-17 |
| Campylobacter | 3.74 | -2.35 | 16-17 |
| Hydrogenoanaerobacterium | 21.04 | 2.82 | 17-18 |
| Ruminococcaceae UCG-008 | 10.47 | 2.52 | 18-19 |
| Ruminococcaceae UCG-004 | 40.26 | 2.15 | 18-19 |
| Oscillospira | 12.08 | 2.56 | 18-19 |
| Intestinimonas | 1028.88 | 2.10 | 19-20 |
| Escherichia-Shigella | 1946.88 | -2.68 | 19-20 |
| Proteus | 4.49 | 2.02 | 20-21 |
| Bacillus | 329.21 | 2.52 | 23-24 |
| Holdemania | 11.81 | 2.29 | 23-24 |
| Faecalibacterium | 8727.64 | 2.15 | 23-24 |
| Oscillibacter | 11.58 | 2.00 | 25-26 |
| Collinsella | 11.63 | -2.31 | 25-26 |
| Lachnospiraceae UCG-006 | 6.87 | 2.46 | 26-27 |
| Erysipelotrichaceae;Dielma | 3.46 | 2.17 | 26-27 |
| Intestinibacter | 22.43 | 2.78 | 27-28 |
| Dielma | 3.57 | -2.30 | 27-28 |
| Lachnospiraceae UCG-006 | 7.16 | -2.41 | 27-28 |
| Faecalitalea | 21.99 | -2.02 | 27-28 |
| Coriobacteriaceae UCG-002 | 25.30 | 3.37 | 28-29 |
| Bacillus | 562.14 | 3.18 | 30-31 |
