# Supplementary material for: Comprehensive Longitudinal Microbiome Analysis of the Chicken Cecum Reveals a Shift From Competitive to Environmental Drivers and a Window of Opportunity for Campylobacter
Source: Front Microbiol. 2018 Oct 15;9:2452. doi: 10.3389/fmicb.2018.02452 (PMC6196313; doi:10.3389/fmicb.2018.02452)
Supplement: Supplementary Table 3 — Differential analysis of pathways becoming significant based on Kruskal-Wallis test (Adjusted P values ≤ 0.05). Here results are shown for both daily and weekly comparisons. [file Table_3.DOCX]

Supplementary Table 3: Differential analysis of pathways becoming significant based on Kruskal-Wallis test (Adjusted P values ≤ 0.05). Here results are shown for both daily and weekly comparisons.

| KEGG KOs | P Values | Adjusted P Values | Upregulated | Group Comparison |
| --- | --- | --- | --- | --- |
| ko00061; Fatty acid biosynthesis | 0.007911789 | 0.037310224 | 03 | 03 - 04 |
| ko00240; Pyrimidine metabolism | 0.009374768 | 0.037310224 | 04 | 03 - 04 |
| ko00250; Alanine, aspartate and glutamate metabolism | 0.006656727 | 0.037310224 | 04 | 03 - 04 |
| ko00290; Valine, leucine and isoleucine biosynthesis | 0.009374768 | 0.037310224 | 04 | 03 - 04 |
| ko00330; Arginine and proline metabolism | 0.009374768 | 0.037310224 | 04 | 03 - 04 |
| ko00350; Tyrosine metabolism | 0.011074438 | 0.037310224 | 04 | 03 - 04 |
| ko00460; Cyanoamino acid metabolism | 0.002213692 | 0.037310224 | 03 | 03 - 04 |
| ko00524; Butirosin and neomycin biosynthesis | 0.009374768 | 0.037310224 | 03 | 03 - 04 |
| ko00562; Inositol phosphate metabolism | 0.006656727 | 0.037310224 | 03 | 03 - 04 |
| ko00624; Polycyclic aromatic hydrocarbon degradation | 0.01304252 | 0.037310224 | 04 | 03 - 04 |
| ko00730; Thiamine metabolism | 0.011074438 | 0.037310224 | 04 | 03 - 04 |
| ko00740; Riboflavin metabolism | 0.01304252 | 0.037310224 | 03 | 03 - 04 |
| ko00750; Vitamin B6 metabolism | 0.007911789 | 0.037310224 | 04 | 03 - 04 |
| ko00770; Pantothenate and CoA biosynthesis | 0.009374768 | 0.037310224 | 04 | 03 - 04 |
| ko00780; Biotin metabolism | 0.01304252 | 0.037310224 | 04 | 03 - 04 |
| ko00906; Carotenoid biosynthesis | 0.01304252 | 0.037310224 | 04 | 03 - 04 |
| ko00960; Tropane, piperidine and pyridine alkaloid biosynthesis | 0.007911789 | 0.037310224 | 03 | 03 - 04 |
| ko01051; Biosynthesis of ansamycins | 0.009374768 | 0.037310224 | 03 | 03 - 04 |
| ko02010; ABC transporters | 0.009374768 | 0.037310224 | 03 | 03 - 04 |
| ko03008; Ribosome biogenesis in eukaryotes | 0.011074438 | 0.037310224 | 03 | 03 - 04 |
| ko03018; RNA degradation | 0.006656727 | 0.037310224 | 03 | 03 - 04 |
| ko03410; Base excision repair | 0.011074438 | 0.037310224 | 03 | 03 - 04 |
| ko04020; Calcium signaling pathway | 0.009374768 | 0.037310224 | 04 | 03 - 04 |
| ko04122; Sulfur relay system | 0.009374768 | 0.037310224 | 04 | 03 - 04 |
| ko04141; Protein processing in endoplasmic reticulum | 0.007911789 | 0.037310224 | 04 | 03 - 04 |
| ko04146; Peroxisome | 0.007911789 | 0.037310224 | 03 | 03 - 04 |
| ko04260; Cardiac muscle contraction | 0.011074438 | 0.037310224 | 03 | 03 - 04 |
| ko04370; VEGF signaling pathway | 0.011074438 | 0.037310224 | 04 | 03 - 04 |
| ko04626; Plant-pathogen interaction | 0.007911789 | 0.037310224 | 03 | 03 - 04 |
| ko04666; Fc gamma R-mediated phagocytosis | 0.009374768 | 0.037310224 | 04 | 03 - 04 |
| ko04728; Dopaminergic synapse | 0.01304252 | 0.037310224 | 03 | 03 - 04 |
| ko04930; Type II diabetes mellitus | 0.011074438 | 0.037310224 | 03 | 03 - 04 |
| ko04974; Protein digestion and absorption | 0.007911789 | 0.037310224 | 03 | 03 - 04 |
| ko05030; Cocaine addiction | 0.005583617 | 0.037310224 | 03 | 03 - 04 |
| ko05031; Amphetamine addiction | 0.005583617 | 0.037310224 | 03 | 03 - 04 |
| ko05034; Alcoholism | 0.01304252 | 0.037310224 | 03 | 03 - 04 |
| ko00190; Oxidative phosphorylation | 0.015313822 | 0.03909974 | 04 | 03 - 04 |
| ko00561; Glycerolipid metabolism | 0.015313822 | 0.03909974 | 03 | 03 - 04 |
| ko04070; Phosphatidylinositol signaling system | 0.015313822 | 0.03909974 | 03 | 03 - 04 |
| ko04112; Cell cycle - Caulobacter | 0.015313822 | 0.03909974 | 04 | 03 - 04 |
| ko04726; Serotonergic synapse | 0.015313822 | 0.03909974 | 03 | 03 - 04 |
| ko05032; Morphine addiction | 0.015313822 | 0.03909974 | 03 | 03 - 04 |
| ko05203; Viral carcinogenesis | 0.015313822 | 0.03909974 | 03 | 03 - 04 |
| ko04144; Endocytosis | 0.000219851 | 0.007285107 | 05 | 05-06 |
| ko04912; GnRH signaling pathway | 0.000219851 | 0.007285107 | 05 | 05-06 |
| ko05012; Parkinsons disease | 0.000219851 | 0.007285107 | 05 | 05-06 |
| ko00030; Pentose phosphate pathway | 0.001822735 | 0.013247142 | 05 | 05-06 |
| ko00053; Ascorbate and aldarate metabolism | 0.002213692 | 0.013247142 | 05 | 05-06 |
| ko00100; Steroid biosynthesis | 0.001822735 | 0.013247142 | 05 | 05-06 |
| ko00196; Photosynthesis - antenna proteins | 0.002208779 | 0.013247142 | 05 | 05-06 |
| ko00540; Lipopolysaccharide biosynthesis | 0.000998686 | 0.013247142 | 05 | 05-06 |
| ko00592; alpha-Linolenic acid metabolism | 0.000275504 | 0.013247142 | 05 | 05-06 |
| ko00623; Toluene degradation | 0.000275504 | 0.013247142 | 05 | 05-06 |
| ko00965; Betalain biosynthesis | 0.000532006 | 0.013247142 | 05 | 05-06 |
| ko00984; Steroid degradation | 0.001496164 | 0.013247142 | 05 | 05-06 |
| ko01057; Biosynthesis of type II polyketide products | 0.000998686 | 0.013247142 | 05 | 05-06 |
| ko02060; Phosphotransferase system (PTS) | 0.001224283 | 0.013247142 | 05 | 05-06 |
| ko04610; Complement and coagulation cascades | 0.001221273 | 0.013247142 | 05 | 05-06 |
| ko05014; Amyotrophic lateral sclerosis (ALS) | 0.000998686 | 0.013247142 | 05 | 05-06 |
| ko05020; Prion diseases | 0.000658337 | 0.013247142 | 05 | 05-06 |
| ko05131; Shigellosis | 0.001221273 | 0.013247142 | 05 | 05-06 |
| ko05142; Chagas disease (American trypanosomiasis) | 0.001822735 | 0.013247142 | 05 | 05-06 |
| ko05204; Chemical carcinogenesis | 0.001224283 | 0.013247142 | 05 | 05-06 |
| ko00363; Bisphenol degradation | 0.002680172 | 0.036672283 | 05 | 05-06 |
| ko00630; Glyoxylate and dicarboxylate metabolism | 0.002680172 | 0.036672283 | 05 | 05-06 |
| ko00901; Indole alkaloid biosynthesis | 0.002680172 | 0.036672283 | 05 | 05-06 |
| ko00920; Sulfur metabolism | 0.002680172 | 0.036672283 | 05 | 05-06 |
| ko00945; Stilbenoid, diarylheptanoid and gingerol biosynthesis | 0.002680172 | 0.036672283 | 05 | 05-06 |
| ko00980; Metabolism of xenobiotics by cytochrome P450 | 0.002680172 | 0.036672283 | 05 | 05-06 |
| ko00982; Drug metabolism - cytochrome P450 | 0.002680172 | 0.036672283 | 05 | 05-06 |
| ko05111; Vibrio cholerae pathogenic cycle | 0.002680172 | 0.036672283 | 05 | 05-06 |
| ko05132; Salmonella infection | 0.002680172 | 0.036672283 | 05 | 05-06 |
| ko04115; p53 signaling pathway | 0.00128048 | 0.028496707 | 10 | 10 -11 |
| ko00909; Sesquiterpenoid and triterpenoid biosynthesis | 0.001702519 | 0.045759699 | 10 | 10 -11 |
| ko05168; Herpes simplex infection | 0.001702519 | 0.045759699 | 10 | 10 -11 |
| ko05210; Colorectal cancer | 0.001702519 | 0.045759699 | 10 | 10 -11 |
| ko05416; Viral myocarditis | 0.001702519 | 0.045759699 | 10 | 10 -11 |
| ko02040; Flagellar assembly | 0.000165522 | 0.012327241 | 14 | 14 - 15 |
| ko05111; Vibrio cholerae pathogenic cycle | 0.000219473 | 0.012327241 | 14 | 14 - 15 |
| ko05130; Pathogenic Escherichia coli infection | 0.000380365 | 0.012327241 | 14 | 14 - 15 |
| ko00364; Fluorobenzoate degradation | 0.001078987 | 0.0146559 | 14 | 14 - 15 |
| ko00592; alpha-Linolenic acid metabolism | 0.001078987 | 0.0146559 | 14 | 14 - 15 |
| ko00633; Nitrotoluene degradation | 0.000646749 | 0.0146559 | 14 | 14 - 15 |
| ko00710; Carbon fixation in photosynthetic organisms | 0.000497167 | 0.0146559 | 15 | 14 - 15 |
| ko03070; Bacterial secretion system | 0.000497167 | 0.0146559 | 14 | 14 - 15 |
| ko05132; Salmonella infection | 0.000497167 | 0.0146559 | 14 | 14 - 15 |
| ko00240; Pyrimidine metabolism | 0.001766337 | 0.01897042 | 15 | 14 - 15 |
| ko00760; Nicotinate and nicotinamide metabolism | 0.001383791 | 0.01897042 | 15 | 14 - 15 |
| ko00920; Sulfur metabolism | 0.001383791 | 0.01897042 | 14 | 14 - 15 |
| ko00960; Tropane, piperidine and pyridine alkaloid biosynthesis | 0.001383791 | 0.01897042 | 14 | 14 - 15 |
| ko02020; Two-component system | 0.001383791 | 0.01897042 | 14 | 14 - 15 |
| ko03440; Homologous recombination | 0.001766337 | 0.01897042 | 15 | 14 - 15 |
| ko05032; Morphine addiction | 0.001383791 | 0.01897042 | 14 | 14 - 15 |
| ko05133; Pertussis | 0.001383791 | 0.01897042 | 14 | 14 - 15 |
| ko00020; Citrate cycle (TCA cycle) | 0.002837545 | 0.022375931 | 14 | 14 - 15 |
| ko00232; Caffeine metabolism | 0.003571236 | 0.022375931 | 14 | 14 - 15 |
| ko00281; Geraniol degradation | 0.003571236 | 0.022375931 | 14 | 14 - 15 |
| ko00310; Lysine degradation | 0.001766337 | 0.022375931 | 14 | 14 - 15 |
| ko00473; D-Alanine metabolism | 0.002837545 | 0.022375931 | 15 | 14 - 15 |
| ko00511; Other glycan degradation | 0.003571236 | 0.022375931 | 15 | 14 - 15 |
| ko00603; Glycosphingolipid biosynthesis - globo series | 0.003571236 | 0.022375931 | 15 | 14 - 15 |
| ko00624; Polycyclic aromatic hydrocarbon degradation | 0.002837545 | 0.022375931 | 15 | 14 - 15 |
| ko00630; Glyoxylate and dicarboxylate metabolism | 0.002837545 | 0.022375931 | 14 | 14 - 15 |
| ko00640; Propanoate metabolism | 0.002244033 | 0.022375931 | 14 | 14 - 15 |
| ko00740; Riboflavin metabolism | 0.003571236 | 0.022375931 | 14 | 14 - 15 |
| ko03030; DNA replication | 0.003571236 | 0.022375931 | 15 | 14 - 15 |
| ko04113; Meiosis - yeast | 0.002244033 | 0.022375931 | 14 | 14 - 15 |
| ko00791; Atrazine degradation | 0.004473649 | 0.041073328 | 14 | 14 - 15 |
| ko00906; Carotenoid biosynthesis | 0.004473649 | 0.041073328 | 15 | 14 - 15 |
| ko00910; Nitrogen metabolism | 0.004473649 | 0.041073328 | 14 | 14 - 15 |
| ko01040; Biosynthesis of unsaturated fatty acids | 0.004473649 | 0.041073328 | 14 | 14 - 15 |
| ko03010; Ribosome | 0.004473649 | 0.041073328 | 15 | 14 - 15 |
| ko05150; Staphylococcus aureus infection | 0.004473649 | 0.041073328 | 15 | 14 - 15 |
| ko00331; Clavulanic acid biosynthesis | 1.16E-09 | 4.38E-09 | Day03-07 | Day03-07 - Day08-14 |
| ko00592; alpha-Linolenic acid metabolism | 1.05E-09 | 4.38E-09 | Day03-07 | Day03-07 - Day08-14 |
| ko00920; Sulfur metabolism | 1.07E-09 | 4.38E-09 | Day03-07 | Day03-07 - Day08-14 |
| ko04011; MAPK signaling pathway - yeast | 1.13E-09 | 4.38E-09 | Day08-14 | Day03-07 - Day08-14 |
| ko04723; Retrograde endocannabinoid signaling | 8.68E-10 | 4.38E-09 | Day03-07 | Day03-07 - Day08-14 |
| ko04726; Serotonergic synapse | 8.91E-10 | 4.38E-09 | Day03-07 | Day03-07 - Day08-14 |
| ko05020; Prion diseases | 4.30E-10 | 4.38E-09 | Day03-07 | Day03-07 - Day08-14 |
| ko05132; Salmonella infection | 2.56E-10 | 4.38E-09 | Day03-07 | Day03-07 - Day08-14 |
| ko05133; Pertussis | 8.01E-10 | 4.38E-09 | Day03-07 | Day03-07 - Day08-14 |
| ko05014; Amyotrophic lateral sclerosis (ALS) | 1.19E-09 | 6.19E-09 | Day03-07 | Day03-07 - Day08-14 |
| ko00380; Tryptophan metabolism | 6.45E-09 | 1.99E-08 | Day03-07 | Day03-07 - Day08-14 |
| ko01053; Biosynthesis of siderophore group nonribosomal peptides | 5.26E-09 | 1.99E-08 | Day03-07 | Day03-07 - Day08-14 |
| ko04144; Endocytosis | 5.98E-09 | 1.99E-08 | Day03-07 | Day03-07 - Day08-14 |
| ko04610; Complement and coagulation cascades | 5.13E-09 | 1.99E-08 | Day03-07 | Day03-07 - Day08-14 |
| ko04912; GnRH signaling pathway | 5.98E-09 | 1.99E-08 | Day03-07 | Day03-07 - Day08-14 |
| ko05034; Alcoholism | 3.15E-09 | 1.99E-08 | Day03-07 | Day03-07 - Day08-14 |
| ko05110; Vibrio cholerae infection | 1.36E-09 | 1.99E-08 | Day03-07 | Day03-07 - Day08-14 |
| ko05111; Vibrio cholerae pathogenic cycle | 4.63E-09 | 1.99E-08 | Day03-07 | Day03-07 - Day08-14 |
| ko05131; Shigellosis | 4.40E-09 | 1.99E-08 | Day03-07 | Day03-07 - Day08-14 |
| ko05219; Bladder cancer | 3.32E-09 | 1.99E-08 | Day03-07 | Day03-07 - Day08-14 |
| ko00030; Pentose phosphate pathway | 2.42E-08 | 8.26E-08 | Day03-07 | Day03-07 - Day08-14 |
| ko00480; Glutathione metabolism | 1.67E-08 | 8.26E-08 | Day03-07 | Day03-07 - Day08-14 |
| ko00540; Lipopolysaccharide biosynthesis | 1.63E-08 | 8.26E-08 | Day03-07 | Day03-07 - Day08-14 |
| ko00562; Inositol phosphate metabolism | 2.73E-08 | 8.26E-08 | Day03-07 | Day03-07 - Day08-14 |
| ko00901; Indole alkaloid biosynthesis | 6.79E-09 | 8.26E-08 | Day03-07 | Day03-07 - Day08-14 |
| ko00965; Betalain biosynthesis | 2.04E-08 | 8.26E-08 | Day03-07 | Day03-07 - Day08-14 |
| ko00982; Drug metabolism - cytochrome P450 | 3.01E-08 | 8.26E-08 | Day03-07 | Day03-07 - Day08-14 |
| ko01040; Biosynthesis of unsaturated fatty acids | 1.15E-08 | 8.26E-08 | Day03-07 | Day03-07 - Day08-14 |
| ko05100; Bacterial invasion of epithelial cells | 2.94E-08 | 8.26E-08 | Day03-07 | Day03-07 - Day08-14 |
| ko05204; Chemical carcinogenesis | 1.21E-08 | 8.26E-08 | Day03-07 | Day03-07 - Day08-14 |
| ko05340; Primary immunodeficiency | 1.84E-08 | 8.26E-08 | Day03-07 | Day03-07 - Day08-14 |
| ko00564; Glycerophospholipid metabolism | 3.16E-08 | 1.95E-07 | Day03-07 | Day03-07 - Day08-14 |
| ko00980; Metabolism of xenobiotics by cytochrome P450 | 4.43E-08 | 1.95E-07 | Day03-07 | Day03-07 - Day08-14 |
| ko03010; Ribosome | 4.02E-08 | 1.95E-07 | Day08-14 | Day03-07 - Day08-14 |
| ko00196; Photosynthesis - antenna proteins | 6.04E-08 | 3.54E-07 | Day03-07 | Day03-07 - Day08-14 |
| ko05142; Chagas disease (American trypanosomiasis) | 4.76E-08 | 3.54E-07 | Day03-07 | Day03-07 - Day08-14 |
| ko05030; Cocaine addiction | 7.66E-08 | 6.61E-07 | Day03-07 | Day03-07 - Day08-14 |
| ko05031; Amphetamine addiction | 7.66E-08 | 6.61E-07 | Day03-07 | Day03-07 - Day08-14 |
| ko00310; Lysine degradation | 1.85E-07 | 1.40E-06 | Day03-07 | Day03-07 - Day08-14 |
| ko00660; C5-Branched dibasic acid metabolism | 2.73E-07 | 1.40E-06 | Day03-07 | Day03-07 - Day08-14 |
| ko00945; Stilbenoid, diarylheptanoid and gingerol biosynthesis | 9.46E-08 | 1.40E-06 | Day03-07 | Day03-07 - Day08-14 |
| ko04728; Dopaminergic synapse | 2.03E-07 | 1.40E-06 | Day03-07 | Day03-07 - Day08-14 |
| ko05012; Parkinsons disease | 2.79E-07 | 1.40E-06 | Day03-07 | Day03-07 - Day08-14 |
| ko00071; Fatty acid metabolism | 1.15E-06 | 4.07E-06 | Day03-07 | Day03-07 - Day08-14 |
| ko00232; Caffeine metabolism | 7.11E-07 | 4.07E-06 | Day03-07 | Day03-07 - Day08-14 |
| ko00363; Bisphenol degradation | 1.68E-06 | 4.07E-06 | Day03-07 | Day03-07 - Day08-14 |
| ko00471; D-Glutamine and D-glutamate metabolism | 1.51E-06 | 4.07E-06 | Day08-14 | Day03-07 - Day08-14 |
| ko00601; Glycosphingolipid biosynthesis - lacto and neolacto series | 9.04E-07 | 4.07E-06 | Day08-14 | Day03-07 - Day08-14 |
| ko00642; Ethylbenzene degradation | 2.86E-07 | 4.07E-06 | Day08-14 | Day03-07 - Day08-14 |
| ko00680; Methane metabolism | 1.42E-06 | 4.07E-06 | Day08-14 | Day03-07 - Day08-14 |
| ko00830; Retinol metabolism | 1.68E-06 | 4.07E-06 | Day03-07 | Day03-07 - Day08-14 |
| ko04727; GABAergic synapse | 1.15E-06 | 4.07E-06 | Day08-14 | Day03-07 - Day08-14 |
| ko04940; Type I diabetes mellitus | 4.09E-07 | 4.07E-06 | Day08-14 | Day03-07 - Day08-14 |
| ko05032; Morphine addiction | 1.05E-06 | 4.07E-06 | Day03-07 | Day03-07 - Day08-14 |
| ko05130; Pathogenic Escherichia coli infection | 9.64E-07 | 4.07E-06 | Day03-07 | Day03-07 - Day08-14 |
| ko05164; Influenza A | 1.76E-06 | 4.07E-06 | Day08-14 | Day03-07 - Day08-14 |
| ko05203; Viral carcinogenesis | 1.83E-06 | 4.07E-06 | Day03-07 | Day03-07 - Day08-14 |
| ko00740; Riboflavin metabolism | 1.95E-06 | 4.65E-06 | Day03-07 | Day03-07 - Day08-14 |
| ko04724; Glutamatergic synapse | 1.95E-06 | 4.65E-06 | Day08-14 | Day03-07 - Day08-14 |
| ko00630; Glyoxylate and dicarboxylate metabolism | 2.08E-06 | 6.01E-06 | Day03-07 | Day03-07 - Day08-14 |
| ko00640; Propanoate metabolism | 2.26E-06 | 6.01E-06 | Day03-07 | Day03-07 - Day08-14 |
| ko04930; Type II diabetes mellitus | 2.41E-06 | 1.82E-05 | Day03-07 | Day03-07 - Day08-14 |
| ko00603; Glycosphingolipid biosynthesis - globo series | 4.56E-06 | 2.31E-05 | Day08-14 | Day03-07 - Day08-14 |
| ko00860; Porphyrin and chlorophyll metabolism | 5.93E-06 | 2.31E-05 | Day03-07 | Day03-07 - Day08-14 |
| ko04146; Peroxisome | 4.65E-06 | 2.31E-05 | Day03-07 | Day03-07 - Day08-14 |
| ko04910; Insulin signaling pathway | 2.90E-06 | 2.31E-05 | Day03-07 | Day03-07 - Day08-14 |
| ko05168; Herpes simplex infection | 5.58E-06 | 2.31E-05 | Day08-14 | Day03-07 - Day08-14 |
| ko00130; Ubiquinone and other terpenoid-quinone biosynthesis | 6.05E-06 | 2.46E-05 | Day03-07 | Day03-07 - Day08-14 |
| ko04070; Phosphatidylinositol signaling system | 6.96E-06 | 2.46E-05 | Day03-07 | Day03-07 - Day08-14 |
| ko00312; beta-Lactam resistance | 1.14E-05 | 2.59E-05 | Day08-14 | Day03-07 - Day08-14 |
| ko00350; Tyrosine metabolism | 9.76E-06 | 2.59E-05 | Day08-14 | Day03-07 - Day08-14 |
| ko00400; Phenylalanine, tyrosine and tryptophan biosynthesis | 1.39E-05 | 2.59E-05 | Day08-14 | Day03-07 - Day08-14 |
| ko00623; Toluene degradation | 1.56E-05 | 2.59E-05 | Day03-07 | Day03-07 - Day08-14 |
| ko00710; Carbon fixation in photosynthetic organisms | 1.44E-05 | 2.59E-05 | Day08-14 | Day03-07 - Day08-14 |
| ko01057; Biosynthesis of type II polyketide products | 1.33E-05 | 2.59E-05 | Day03-07 | Day03-07 - Day08-14 |
| ko02060; Phosphotransferase system (PTS) | 7.39E-06 | 2.59E-05 | Day03-07 | Day03-07 - Day08-14 |
| ko03008; Ribosome biogenesis in eukaryotes | 1.47E-05 | 2.59E-05 | Day03-07 | Day03-07 - Day08-14 |
| ko03018; RNA degradation | 1.50E-05 | 2.59E-05 | Day03-07 | Day03-07 - Day08-14 |
| ko03060; Protein export | 1.44E-05 | 2.59E-05 | Day08-14 | Day03-07 - Day08-14 |
| ko05210; Colorectal cancer | 1.16E-05 | 2.59E-05 | Day08-14 | Day03-07 - Day08-14 |
| ko05416; Viral myocarditis | 1.16E-05 | 2.59E-05 | Day08-14 | Day03-07 - Day08-14 |
| ko00061; Fatty acid biosynthesis | 1.59E-05 | 3.45E-05 | Day03-07 | Day03-07 - Day08-14 |
| ko00100; Steroid biosynthesis | 1.68E-05 | 8.68E-05 | Day03-07 | Day03-07 - Day08-14 |
| ko00340; Histidine metabolism | 1.96E-05 | 8.68E-05 | Day08-14 | Day03-07 - Day08-14 |
| ko00401; Novobiocin biosynthesis | 2.16E-05 | 0.000109709 | Day08-14 | Day03-07 - Day08-14 |
| ko00621; Dioxin degradation | 2.51E-05 | 0.000109709 | Day08-14 | Day03-07 - Day08-14 |
| ko00010; Glycolysis / Gluconeogenesis | 6.52E-05 | 0.000145793 | Day03-07 | Day03-07 - Day08-14 |
| ko00053; Ascorbate and aldarate metabolism | 0.000110879 | 0.000145793 | Day03-07 | Day03-07 - Day08-14 |
| ko00140; Steroid hormone biosynthesis | 2.75E-05 | 0.000145793 | Day03-07 | Day03-07 - Day08-14 |
| ko00361; Chlorocyclohexane and chlorobenzene degradation | 7.13E-05 | 0.000145793 | Day03-07 | Day03-07 - Day08-14 |
| ko00430; Taurine and hypotaurine metabolism | 0.000120947 | 0.000145793 | Day03-07 | Day03-07 - Day08-14 |
| ko00524; Butirosin and neomycin biosynthesis | 9.31E-05 | 0.000145793 | Day03-07 | Day03-07 - Day08-14 |
| ko00565; Ether lipid metabolism | 8.52E-05 | 0.000145793 | Day03-07 | Day03-07 - Day08-14 |
| ko00970; Aminoacyl-tRNA biosynthesis | 0.000114808 | 0.000145793 | Day08-14 | Day03-07 - Day08-14 |
| ko02040; Flagellar assembly | 3.26E-05 | 0.000145793 | Day03-07 | Day03-07 - Day08-14 |
| ko03030; DNA replication | 0.000107078 | 0.000145793 | Day08-14 | Day03-07 - Day08-14 |
| ko03450; Non-homologous end-joining | 7.53E-05 | 0.000145793 | Day03-07 | Day03-07 - Day08-14 |
| ko04310; Wnt signaling pathway | 8.98E-05 | 0.000145793 | Day08-14 | Day03-07 - Day08-14 |
| ko04330; Notch signaling pathway | 8.98E-05 | 0.000145793 | Day08-14 | Day03-07 - Day08-14 |
| ko04514; Cell adhesion molecules (CAMs) | 6.25E-05 | 0.000145793 | Day08-14 | Day03-07 - Day08-14 |
| ko04640; Hematopoietic cell lineage | 6.76E-05 | 0.000145793 | Day08-14 | Day03-07 - Day08-14 |
| ko04721; Synaptic vesicle cycle | 0.000108963 | 0.000145793 | Day08-14 | Day03-07 - Day08-14 |
| ko04725; Cholinergic synapse | 6.41E-05 | 0.000145793 | Day03-07 | Day03-07 - Day08-14 |
| ko04810; Regulation of actin cytoskeleton | 0.000118694 | 0.000145793 | Day08-14 | Day03-07 - Day08-14 |
| ko04962; Vasopressin-regulated water reabsorption | 0.000108963 | 0.000145793 | Day08-14 | Day03-07 - Day08-14 |
| ko04975; Fat digestion and absorption | 8.22E-05 | 0.000145793 | Day08-14 | Day03-07 - Day08-14 |
| ko05202; Transcriptional misregulation in cancer | 9.32E-05 | 0.000145793 | Day08-14 | Day03-07 - Day08-14 |
| ko05220; Chronic myeloid leukemia | 8.98E-05 | 0.000145793 | Day08-14 | Day03-07 - Day08-14 |
| ko05412; Arrhythmogenic right ventricular cardiomyopathy (ARVC) | 6.25E-05 | 0.000145793 | Day08-14 | Day03-07 - Day08-14 |
| ko05414; Dilated cardiomyopathy | 6.25E-05 | 0.000145793 | Day08-14 | Day03-07 - Day08-14 |
| ko00450; Selenocompound metabolism | 0.000131872 | 0.000236604 | Day03-07 | Day03-07 - Day08-14 |
| ko00604; Glycosphingolipid biosynthesis - ganglio series | 0.000123061 | 0.000236604 | Day08-14 | Day03-07 - Day08-14 |
| ko00240; Pyrimidine metabolism | 0.000215926 | 0.000470296 | Day08-14 | Day03-07 - Day08-14 |
| ko01055; Biosynthesis of vancomycin group antibiotics | 0.000219572 | 0.000470296 | Day08-14 | Day03-07 - Day08-14 |
| ko02010; ABC transporters | 0.00017638 | 0.000470296 | Day03-07 | Day03-07 - Day08-14 |
| ko04064; NF-kappa B signaling pathway | 0.000233992 | 0.000470296 | Day08-14 | Day03-07 - Day08-14 |
| ko04112; Cell cycle - Caulobacter | 0.000191949 | 0.000470296 | Day08-14 | Day03-07 - Day08-14 |
| ko04626; Plant-pathogen interaction | 0.000167617 | 0.000470296 | Day03-07 | Day03-07 - Day08-14 |
| ko04916; Melanogenesis | 0.000135979 | 0.000470296 | Day08-14 | Day03-07 - Day08-14 |
| ko05140; Leishmaniasis | 0.000233992 | 0.000470296 | Day08-14 | Day03-07 - Day08-14 |
| ko00253; Tetracycline biosynthesis | 0.000277087 | 0.000568971 | Day03-07 | Day03-07 - Day08-14 |
| ko00785; Lipoic acid metabolism | 0.000300849 | 0.000568971 | Day03-07 | Day03-07 - Day08-14 |
| ko03070; Bacterial secretion system | 0.000268084 | 0.000568971 | Day03-07 | Day03-07 - Day08-14 |
| ko03440; Homologous recombination | 0.000238688 | 0.000568971 | Day08-14 | Day03-07 - Day08-14 |
| ko04080; Neuroactive ligand-receptor interaction | 0.000292503 | 0.000568971 | Day08-14 | Day03-07 - Day08-14 |
| ko04622; RIG-I-like receptor signaling pathway | 0.000321219 | 0.000568971 | Day03-07 | Day03-07 - Day08-14 |
| ko00300; Lysine biosynthesis | 0.000331881 | 0.000834975 | Day08-14 | Day03-07 - Day08-14 |
| ko00364; Fluorobenzoate degradation | 0.00038409 | 0.000834975 | Day03-07 | Day03-07 - Day08-14 |
| ko00720; Carbon fixation pathways in prokaryotes | 0.000360004 | 0.000834975 | Day08-14 | Day03-07 - Day08-14 |
| ko05169; Epstein-Barr virus infection | 0.000419343 | 0.000834975 | Day03-07 | Day03-07 - Day08-14 |
| ko04141; Protein processing in endoplasmic reticulum | 0.000423057 | 0.002396589 | Day08-14 | Day03-07 - Day08-14 |
| ko00460; Cyanoamino acid metabolism | 0.000590446 | 0.002834065 | Day03-07 | Day03-07 - Day08-14 |
| ko00523; Polyketide sugar unit biosynthesis | 0.000700997 | 0.002834065 | Day08-14 | Day03-07 - Day08-14 |
| ko04122; Sulfur relay system | 0.00074576 | 0.002834065 | Day08-14 | Day03-07 - Day08-14 |
| ko05016; Huntingtons disease | 0.000856352 | 0.002834065 | Day03-07 | Day03-07 - Day08-14 |
| ko05134; Legionellosis | 0.000488481 | 0.002834065 | Day08-14 | Day03-07 - Day08-14 |
| ko05200; Pathways in cancer | 0.000896474 | 0.002834065 | Day08-14 | Day03-07 - Day08-14 |
| ko00250; Alanine, aspartate and glutamate metabolism | 0.001324644 | 0.005498727 | Day08-14 | Day03-07 - Day08-14 |
| ko00730; Thiamine metabolism | 0.001364383 | 0.005498727 | Day08-14 | Day03-07 - Day08-14 |
| ko00940; Phenylpropanoid biosynthesis | 0.001826811 | 0.005498727 | Day03-07 | Day03-07 - Day08-14 |
| ko05120; Epithelial cell signaling in Helicobacter pylori infection | 0.001468591 | 0.005498727 | Day03-07 | Day03-07 - Day08-14 |
| ko05211; Renal cell carcinoma | 0.001774802 | 0.005498727 | Day08-14 | Day03-07 - Day08-14 |
| ko05222; Small cell lung cancer | 0.000952682 | 0.005498727 | Day08-14 | Day03-07 - Day08-14 |
| ko00020; Citrate cycle (TCA cycle) | 0.001853337 | 0.005535155 | Day03-07 | Day03-07 - Day08-14 |
| ko00195; Photosynthesis | 0.006251038 | 0.005535155 | Day08-14 | Day03-07 - Day08-14 |
| ko00330; Arginine and proline metabolism | 0.002463965 | 0.005535155 | Day08-14 | Day03-07 - Day08-14 |
| ko00351; DDT degradation | 0.00279471 | 0.005535155 | Day08-14 | Day03-07 - Day08-14 |
| ko00472; D-Arginine and D-ornithine metabolism | 0.003344319 | 0.005535155 | Day03-07 | Day03-07 - Day08-14 |
| ko00561; Glycerolipid metabolism | 0.002913663 | 0.005535155 | Day03-07 | Day03-07 - Day08-14 |
| ko00591; Linoleic acid metabolism | 0.00481191 | 0.005535155 | Day03-07 | Day03-07 - Day08-14 |
| ko00600; Sphingolipid metabolism | 0.003390427 | 0.005535155 | Day08-14 | Day03-07 - Day08-14 |
| ko00620; Pyruvate metabolism | 0.00415511 | 0.005535155 | Day03-07 | Day03-07 - Day08-14 |
| ko00622; Xylene degradation | 0.00514041 | 0.005535155 | Day08-14 | Day03-07 - Day08-14 |
| ko00624; Polycyclic aromatic hydrocarbon degradation | 0.003209399 | 0.005535155 | Day08-14 | Day03-07 - Day08-14 |
| ko00633; Nitrotoluene degradation | 0.006581604 | 0.005535155 | Day03-07 | Day03-07 - Day08-14 |
| ko00750; Vitamin B6 metabolism | 0.004268115 | 0.005535155 | Day08-14 | Day03-07 - Day08-14 |
| ko00770; Pantothenate and CoA biosynthesis | 0.002395511 | 0.005535155 | Day08-14 | Day03-07 - Day08-14 |
| ko00906; Carotenoid biosynthesis | 0.004211269 | 0.005535155 | Day08-14 | Day03-07 - Day08-14 |
| ko01051; Biosynthesis of ansamycins | 0.002498859 | 0.005535155 | Day03-07 | Day03-07 - Day08-14 |
| ko03015; mRNA surveillance pathway | 0.006251038 | 0.005535155 | Day03-07 | Day03-07 - Day08-14 |
| ko03050; Proteasome | 0.003580706 | 0.005535155 | Day08-14 | Day03-07 - Day08-14 |
| ko04020; Calcium signaling pathway | 0.004502523 | 0.005535155 | Day08-14 | Day03-07 - Day08-14 |
| ko04114; Oocyte meiosis | 0.006799255 | 0.005535155 | Day08-14 | Day03-07 - Day08-14 |
| ko04270; Vascular smooth muscle contraction | 0.006799255 | 0.005535155 | Day08-14 | Day03-07 - Day08-14 |
| ko04370; VEGF signaling pathway | 0.002913663 | 0.005535155 | Day08-14 | Day03-07 - Day08-14 |
| ko04666; Fc gamma R-mediated phagocytosis | 0.004099632 | 0.005535155 | Day08-14 | Day03-07 - Day08-14 |
| ko04720; Long-term potentiation | 0.006799255 | 0.005535155 | Day08-14 | Day03-07 - Day08-14 |
| ko04722; Neurotrophin signaling pathway | 0.006799255 | 0.005535155 | Day08-14 | Day03-07 - Day08-14 |
| ko04740; Olfactory transduction | 0.006799255 | 0.005535155 | Day08-14 | Day03-07 - Day08-14 |
| ko04744; Phototransduction | 0.006799255 | 0.005535155 | Day08-14 | Day03-07 - Day08-14 |
| ko04745; Phototransduction - fly | 0.006799255 | 0.005535155 | Day08-14 | Day03-07 - Day08-14 |
| ko05150; Staphylococcus aureus infection | 0.002078632 | 0.005535155 | Day08-14 | Day03-07 - Day08-14 |
| ko05152; Tuberculosis | 0.006332247 | 0.005535155 | Day08-14 | Day03-07 - Day08-14 |
| ko05160; Hepatitis C | 0.005480406 | 0.005535155 | Day08-14 | Day03-07 - Day08-14 |
| ko05162; Measles | 0.005480406 | 0.005535155 | Day08-14 | Day03-07 - Day08-14 |
| ko05214; Glioma | 0.006799255 | 0.005535155 | Day08-14 | Day03-07 - Day08-14 |
| ko00040; Pentose and glucuronate interconversions | 0.007965754 | 0.009152701 | Day03-07 | Day03-07 - Day08-14 |
| ko00473; D-Alanine metabolism | 0.007865947 | 0.009152701 | Day08-14 | Day03-07 - Day08-14 |
| ko00960; Tropane, piperidine and pyridine alkaloid biosynthesis | 0.006839757 | 0.009152701 | Day03-07 | Day03-07 - Day08-14 |
| ko04961; Endocrine and other factor-regulated calcium reabsorption | 0.007965754 | 0.009152701 | Day08-14 | Day03-07 - Day08-14 |
| ko04972; Pancreatic secretion | 0.007290285 | 0.009152701 | Day08-14 | Day03-07 - Day08-14 |
| ko00052; Galactose metabolism | 0.014999488 | 0.030178743 | Day03-07 | Day03-07 - Day08-14 |
| ko00190; Oxidative phosphorylation | 0.012553962 | 0.030178743 | Day08-14 | Day03-07 - Day08-14 |
| ko00670; One carbon pool by folate | 0.016468133 | 0.030178743 | Day08-14 | Day03-07 - Day08-14 |
| ko00780; Biotin metabolism | 0.016660318 | 0.030178743 | Day08-14 | Day03-07 - Day08-14 |
| ko02030; Bacterial chemotaxis | 0.017448893 | 0.030178743 | Day08-14 | Day03-07 - Day08-14 |
| ko03410; Base excision repair | 0.016089595 | 0.030178743 | Day03-07 | Day03-07 - Day08-14 |
| ko04210; Apoptosis | 0.013486272 | 0.030178743 | Day08-14 | Day03-07 - Day08-14 |
| ko04612; Antigen processing and presentation | 0.014309398 | 0.030178743 | Day03-07 | Day03-07 - Day08-14 |
| ko04914; Progesterone-mediated oocyte maturation | 0.014309398 | 0.030178743 | Day03-07 | Day03-07 - Day08-14 |
| ko04960; Aldosterone-regulated sodium reabsorption | 0.008588768 | 0.030178743 | Day08-14 | Day03-07 - Day08-14 |
| ko04970; Salivary secretion | 0.008588768 | 0.030178743 | Day08-14 | Day03-07 - Day08-14 |
| ko04971; Gastric acid secretion | 0.008588768 | 0.030178743 | Day08-14 | Day03-07 - Day08-14 |
| ko05215; Prostate cancer | 0.014309398 | 0.030178743 | Day03-07 | Day03-07 - Day08-14 |
| ko05014; Amyotrophic lateral sclerosis (ALS) | 3.10E-09 | 2.80E-07 | Day08-14 | Day08-14 - Day15-24 |
| ko03050; Proteasome | 4.41E-09 | 3.83E-07 | Day15-24 | Day08-14 - Day15-24 |
| ko00331; Clavulanic acid biosynthesis | 7.43E-09 | 4.28E-07 | Day08-14 | Day08-14 - Day15-24 |
| ko00592; alpha-Linolenic acid metabolism | 1.77E-08 | 4.28E-07 | Day08-14 | Day08-14 - Day15-24 |
| ko04723; Retrograde endocannabinoid signaling | 1.34E-08 | 4.28E-07 | Day08-14 | Day08-14 - Day15-24 |
| ko05020; Prion diseases | 1.56E-08 | 4.28E-07 | Day08-14 | Day08-14 - Day15-24 |
| ko04974; Protein digestion and absorption | 2.09E-08 | 3.33E-06 | Day15-24 | Day08-14 - Day15-24 |
| ko00380; Tryptophan metabolism | 4.72E-08 | 4.96E-06 | Day08-14 | Day08-14 - Day15-24 |
| ko00400; Phenylalanine, tyrosine and tryptophan biosynthesis | 5.90E-07 | 1.25E-05 | Day15-24 | Day08-14 - Day15-24 |
| ko03040; Spliceosome | 7.74E-07 | 1.25E-05 | Day08-14 | Day08-14 - Day15-24 |
| ko04514; Cell adhesion molecules (CAMs) | 7.77E-07 | 1.25E-05 | Day15-24 | Day08-14 - Day15-24 |
| ko04610; Complement and coagulation cascades | 2.49E-07 | 1.25E-05 | Day08-14 | Day08-14 - Day15-24 |
| ko04724; Glutamatergic synapse | 9.61E-07 | 1.25E-05 | Day15-24 | Day08-14 - Day15-24 |
| ko04810; Regulation of actin cytoskeleton | 6.82E-07 | 1.25E-05 | Day15-24 | Day08-14 - Day15-24 |
| ko05131; Shigellosis | 8.63E-08 | 1.25E-05 | Day08-14 | Day08-14 - Day15-24 |
| ko05412; Arrhythmogenic right ventricular cardiomyopathy (ARVC) | 7.77E-07 | 1.25E-05 | Day15-24 | Day08-14 - Day15-24 |
| ko05414; Dilated cardiomyopathy | 7.77E-07 | 1.25E-05 | Day15-24 | Day08-14 - Day15-24 |
| ko00520; Amino sugar and nucleotide sugar metabolism | 9.74E-07 | 7.45E-05 | Day15-24 | Day08-14 - Day15-24 |
| ko00471; D-Glutamine and D-glutamate metabolism | 4.59E-06 | 0.000191679 | Day15-24 | Day08-14 - Day15-24 |
| ko04916; Melanogenesis | 1.56E-06 | 0.000191679 | Day15-24 | Day08-14 - Day15-24 |
| ko05202; Transcriptional misregulation in cancer | 4.37E-06 | 0.000191679 | Day15-24 | Day08-14 - Day15-24 |
| ko04080; Neuroactive ligand-receptor interaction | 1.26E-05 | 0.000351318 | Day15-24 | Day08-14 - Day15-24 |
| ko04975; Fat digestion and absorption | 1.26E-05 | 0.000351318 | Day15-24 | Day08-14 - Day15-24 |
| ko05169; Epstein-Barr virus infection | 6.10E-06 | 0.000351318 | Day15-24 | Day08-14 - Day15-24 |
| ko00232; Caffeine metabolism | 7.33E-05 | 0.000771811 | Day08-14 | Day08-14 - Day15-24 |
| ko00253; Tetracycline biosynthesis | 4.09E-05 | 0.000771811 | Day08-14 | Day08-14 - Day15-24 |
| ko00401; Novobiocin biosynthesis | 7.75E-05 | 0.000771811 | Day15-24 | Day08-14 - Day15-24 |
| ko00500; Starch and sucrose metabolism | 6.78E-05 | 0.000771811 | Day15-24 | Day08-14 - Day15-24 |
| ko00513; Various types of N-glycan biosynthesis | 1.44E-05 | 0.000771811 | Day15-24 | Day08-14 - Day15-24 |
| ko00623; Toluene degradation | 7.75E-05 | 0.000771811 | Day08-14 | Day08-14 - Day15-24 |
| ko01053; Biosynthesis of siderophore group nonribosomal peptides | 6.49E-05 | 0.000771811 | Day08-14 | Day08-14 - Day15-24 |
| ko01057; Biosynthesis of type II polyketide products | 5.80E-05 | 0.000771811 | Day08-14 | Day08-14 - Day15-24 |
| ko03010; Ribosome | 5.13E-05 | 0.000771811 | Day15-24 | Day08-14 - Day15-24 |
| ko04640; Hematopoietic cell lineage | 2.40E-05 | 0.000771811 | Day15-24 | Day08-14 - Day15-24 |
| ko05100; Bacterial invasion of epithelial cells | 2.93E-05 | 0.000771811 | Day08-14 | Day08-14 - Day15-24 |
| ko00310; Lysine degradation | 0.000145273 | 0.001828317 | Day08-14 | Day08-14 - Day15-24 |
| ko00603; Glycosphingolipid biosynthesis - globo series | 8.74E-05 | 0.001828317 | Day15-24 | Day08-14 - Day15-24 |
| ko00984; Steroid degradation | 0.000156489 | 0.001828317 | Day08-14 | Day08-14 - Day15-24 |
| ko01055; Biosynthesis of vancomycin group antibiotics | 8.74E-05 | 0.001828317 | Day15-24 | Day08-14 - Day15-24 |
| ko05130; Pathogenic Escherichia coli infection | 0.000136266 | 0.001828317 | Day08-14 | Day08-14 - Day15-24 |
| ko05164; Influenza A | 0.000161541 | 0.001828317 | Day15-24 | Day08-14 - Day15-24 |
| ko05204; Chemical carcinogenesis | 0.000163258 | 0.001828317 | Day08-14 | Day08-14 - Day15-24 |
| ko00363; Bisphenol degradation | 0.000173932 | 0.002295258 | Day08-14 | Day08-14 - Day15-24 |
| ko00473; D-Alanine metabolism | 0.000221233 | 0.002295258 | Day15-24 | Day08-14 - Day15-24 |
| ko00901; Indole alkaloid biosynthesis | 0.000203556 | 0.002295258 | Day08-14 | Day08-14 - Day15-24 |
| ko00051; Fructose and mannose metabolism | 0.000564731 | 0.009251318 | Day15-24 | Day08-14 - Day15-24 |
| ko00531; Glycosaminoglycan degradation | 0.000228226 | 0.009251318 | Day15-24 | Day08-14 - Day15-24 |
| ko03015; mRNA surveillance pathway | 0.00050698 | 0.009251318 | Day08-14 | Day08-14 - Day15-24 |
| ko04727; GABAergic synapse | 0.000323394 | 0.009251318 | Day15-24 | Day08-14 - Day15-24 |
| ko05111; Vibrio cholerae pathogenic cycle | 0.000383863 | 0.009251318 | Day08-14 | Day08-14 - Day15-24 |
| ko04064; NF-kappa B signaling pathway | 0.001256247 | 0.017059387 | Day15-24 | Day08-14 - Day15-24 |
| ko04940; Type I diabetes mellitus | 0.000887451 | 0.017059387 | Day15-24 | Day08-14 - Day15-24 |
| ko05110; Vibrio cholerae infection | 0.001041361 | 0.017059387 | Day08-14 | Day08-14 - Day15-24 |
| ko05140; Leishmaniasis | 0.001256247 | 0.017059387 | Day15-24 | Day08-14 - Day15-24 |
| ko05145; Toxoplasmosis | 0.000592958 | 0.017059387 | Day15-24 | Day08-14 - Day15-24 |
| ko00982; Drug metabolism - cytochrome P450 | 0.001465349 | 0.017880782 | Day08-14 | Day08-14 - Day15-24 |
| ko05133; Pertussis | 0.001265523 | 0.017880782 | Day08-14 | Day08-14 - Day15-24 |
| ko00590; Arachidonic acid metabolism | 0.001547502 | 0.018567059 | Day08-14 | Day08-14 - Day15-24 |
| ko00071; Fatty acid metabolism | 0.001954847 | 0.020888313 | Day08-14 | Day08-14 - Day15-24 |
| ko00600; Sphingolipid metabolism | 0.0025238 | 0.020888313 | Day15-24 | Day08-14 - Day15-24 |
| ko00627; Aminobenzoate degradation | 0.002501893 | 0.020888313 | Day08-14 | Day08-14 - Day15-24 |
| ko00790; Folate biosynthesis | 0.00248016 | 0.020888313 | Day15-24 | Day08-14 - Day15-24 |
| ko00980; Metabolism of xenobiotics by cytochrome P450 | 0.001693903 | 0.020888313 | Day08-14 | Day08-14 - Day15-24 |
| ko05012; Parkinsons disease | 0.002545882 | 0.020888313 | Day08-14 | Day08-14 - Day15-24 |
| ko00965; Betalain biosynthesis | 0.002682127 | 0.024353449 | Day08-14 | Day08-14 - Day15-24 |
| ko00960; Tropane, piperidine and pyridine alkaloid biosynthesis | 0.003468705 | 0.045274535 | Day08-14 | Day08-14 - Day15-24 |
| ko05132; Salmonella infection | 0.002874153 | 0.045274535 | Day08-14 | Day08-14 - Day15-24 |
| ko00340; Histidine metabolism | 1.31E-09 | 1.21E-07 | Day15-24 | Day15-24 - Day25-35 |
| ko00030; Pentose phosphate pathway | 2.08E-08 | 1.16E-06 | Day25-35 | Day15-24 - Day25-35 |
| ko04622; RIG-I-like receptor signaling pathway | 2.16E-09 | 1.16E-06 | Day25-35 | Day15-24 - Day25-35 |
| ko05168; Herpes simplex infection | 2.35E-08 | 1.16E-06 | Day15-24 | Day15-24 - Day25-35 |
| ko00710; Carbon fixation in photosynthetic organisms | 3.31E-08 | 1.46E-06 | Day25-35 | Day15-24 - Day25-35 |
| ko00790; Folate biosynthesis | 6.64E-08 | 1.46E-06 | Day25-35 | Day15-24 - Day25-35 |
| ko00010; Glycolysis / Gluconeogenesis | 1.08E-07 | 3.74E-06 | Day25-35 | Day15-24 - Day25-35 |
| ko00521; Streptomycin biosynthesis | 2.34E-07 | 3.74E-06 | Day25-35 | Day15-24 - Day25-35 |
| ko04112; Cell cycle - Caulobacter | 1.82E-07 | 3.74E-06 | Day15-24 | Day15-24 - Day25-35 |
| ko04115; p53 signaling pathway | 1.82E-07 | 3.74E-06 | Day15-24 | Day15-24 - Day25-35 |
| ko05164; Influenza A | 2.56E-07 | 3.74E-06 | Day25-35 | Day15-24 - Day25-35 |
| ko05210; Colorectal cancer | 7.21E-08 | 3.74E-06 | Day15-24 | Day15-24 - Day25-35 |
| ko05416; Viral myocarditis | 7.21E-08 | 3.74E-06 | Day15-24 | Day15-24 - Day25-35 |
| ko02030; Bacterial chemotaxis | 2.72E-07 | 1.40E-05 | Day15-24 | Day15-24 - Day25-35 |
| ko04210; Apoptosis | 5.01E-07 | 1.40E-05 | Day15-24 | Day15-24 - Day25-35 |
| ko00472; D-Arginine and D-ornithine metabolism | 5.21E-07 | 3.11E-05 | Day25-35 | Day15-24 - Day25-35 |
| ko00941; Flavonoid biosynthesis | 7.97E-07 | 3.20E-05 | Day15-24 | Day15-24 - Day25-35 |
| ko00312; beta-Lactam resistance | 5.76E-06 | 0.000163818 | Day15-24 | Day15-24 - Day25-35 |
| ko00523; Polyketide sugar unit biosynthesis | 2.65E-06 | 0.000163818 | Day25-35 | Day15-24 - Day25-35 |
| ko02020; Two-component system | 1.25E-05 | 0.000163818 | Day15-24 | Day15-24 - Day25-35 |
| ko02060; Phosphotransferase system (PTS) | 1.08E-06 | 0.000163818 | Day25-35 | Day15-24 - Day25-35 |
| ko03010; Ribosome | 1.17E-05 | 0.000163818 | Day25-35 | Day15-24 - Day25-35 |
| ko04940; Type I diabetes mellitus | 6.13E-06 | 0.000163818 | Day25-35 | Day15-24 - Day25-35 |
| ko05219; Bladder cancer | 1.06E-05 | 0.000163818 | Day25-35 | Day15-24 - Day25-35 |
| ko05222; Small cell lung cancer | 7.00E-06 | 0.000163818 | Day15-24 | Day15-24 - Day25-35 |
| ko00052; Galactose metabolism | 2.38E-05 | 0.000392037 | Day25-35 | Day15-24 - Day25-35 |
| ko00130; Ubiquinone and other terpenoid-quinone biosynthesis | 1.27E-05 | 0.000392037 | Day25-35 | Day15-24 - Day25-35 |
| ko00290; Valine, leucine and isoleucine biosynthesis | 3.03E-05 | 0.000392037 | Day15-24 | Day15-24 - Day25-35 |
| ko03450; Non-homologous end-joining | 2.70E-05 | 0.000392037 | Day25-35 | Day15-24 - Day25-35 |
| ko04070; Phosphatidylinositol signaling system | 1.69E-05 | 0.000392037 | Day25-35 | Day15-24 - Day25-35 |
| ko05146; Amoebiasis | 2.79E-05 | 0.000392037 | Day15-24 | Day15-24 - Day25-35 |
| ko00604; Glycosphingolipid biosynthesis - ganglio series | 5.21E-05 | 0.001884939 | Day15-24 | Day15-24 - Day25-35 |
| ko04930; Type II diabetes mellitus | 3.37E-05 | 0.001884939 | Day25-35 | Day15-24 - Day25-35 |
| ko00053; Ascorbate and aldarate metabolism | 0.000196433 | 0.00197874 | Day25-35 | Day15-24 - Day25-35 |
| ko00072; Synthesis and degradation of ketone bodies | 0.000154622 | 0.00197874 | Day15-24 | Day15-24 - Day25-35 |
| ko00196; Photosynthesis - antenna proteins | 0.000150028 | 0.00197874 | Day25-35 | Day15-24 - Day25-35 |
| ko00350; Tyrosine metabolism | 0.000159347 | 0.00197874 | Day15-24 | Day15-24 - Day25-35 |
| ko00450; Selenocompound metabolism | 0.000179642 | 0.00197874 | Day25-35 | Day15-24 - Day25-35 |
| ko00471; D-Glutamine and D-glutamate metabolism | 8.71E-05 | 0.00197874 | Day25-35 | Day15-24 - Day25-35 |
| ko00909; Sesquiterpenoid and triterpenoid biosynthesis | 0.00021469 | 0.00197874 | Day15-24 | Day15-24 - Day25-35 |
| ko03430; Mismatch repair | 0.000109797 | 0.00197874 | Day15-24 | Day15-24 - Day25-35 |
| ko04974; Protein digestion and absorption | 6.72E-05 | 0.00197874 | Day25-35 | Day15-24 - Day25-35 |
| ko05142; Chagas disease (American trypanosomiasis) | 0.000241519 | 0.00197874 | Day25-35 | Day15-24 - Day25-35 |
| ko00524; Butirosin and neomycin biosynthesis | 0.000243296 | 0.002969885 | Day25-35 | Day15-24 - Day25-35 |
| ko04310; Wnt signaling pathway | 0.000347041 | 0.002969885 | Day15-24 | Day15-24 - Day25-35 |
| ko04330; Notch signaling pathway | 0.000347041 | 0.002969885 | Day15-24 | Day15-24 - Day25-35 |
| ko05203; Viral carcinogenesis | 0.000367481 | 0.002969885 | Day25-35 | Day15-24 - Day25-35 |
| ko05220; Chronic myeloid leukemia | 0.000347041 | 0.002969885 | Day15-24 | Day15-24 - Day25-35 |
| ko00061; Fatty acid biosynthesis | 0.000605033 | 0.004279392 | Day15-24 | Day15-24 - Day25-35 |
| ko00195; Photosynthesis | 0.000661672 | 0.004279392 | Day25-35 | Day15-24 - Day25-35 |
| ko00253; Tetracycline biosynthesis | 0.000375432 | 0.004279392 | Day15-24 | Day15-24 - Day25-35 |
| ko00540; Lipopolysaccharide biosynthesis | 0.00052298 | 0.004279392 | Day25-35 | Day15-24 - Day25-35 |
| ko00630; Glyoxylate and dicarboxylate metabolism | 0.000661672 | 0.004279392 | Day25-35 | Day15-24 - Day25-35 |
| ko01051; Biosynthesis of ansamycins | 0.000657146 | 0.004279392 | Day25-35 | Day15-24 - Day25-35 |
| ko03060; Protein export | 0.000605033 | 0.004279392 | Day25-35 | Day15-24 - Day25-35 |
| ko05322; Systemic lupus erythematosus | 0.000541518 | 0.004279392 | Day15-24 | Day15-24 - Day25-35 |
| ko00051; Fructose and mannose metabolism | 0.001580412 | 0.011322937 | Day25-35 | Day15-24 - Day25-35 |
| ko00230; Purine metabolism | 0.001388759 | 0.011322937 | Day25-35 | Day15-24 - Day25-35 |
| ko00281; Geraniol degradation | 0.000934912 | 0.011322937 | Day15-24 | Day15-24 - Day25-35 |
| ko00331; Clavulanic acid biosynthesis | 0.001663673 | 0.011322937 | Day25-35 | Day15-24 - Day25-35 |
| ko00351; DDT degradation | 0.001251127 | 0.011322937 | Day15-24 | Day15-24 - Day25-35 |
| ko00362; Benzoate degradation | 0.001962938 | 0.011322937 | Day15-24 | Day15-24 - Day25-35 |
| ko00562; Inositol phosphate metabolism | 0.001026433 | 0.011322937 | Day25-35 | Day15-24 - Day25-35 |
| ko00623; Toluene degradation | 0.002000478 | 0.011322937 | Day25-35 | Day15-24 - Day25-35 |
| ko01055; Biosynthesis of vancomycin group antibiotics | 0.000680068 | 0.011322937 | Day25-35 | Day15-24 - Day25-35 |
| ko01056; Biosynthesis of type II polyketide backbone | 0.002064511 | 0.011322937 | Day15-24 | Day15-24 - Day25-35 |
| ko02010; ABC transporters | 0.001111408 | 0.011322937 | Day25-35 | Day15-24 - Day25-35 |
| ko03008; Ribosome biogenesis in eukaryotes | 0.001762155 | 0.011322937 | Day25-35 | Day15-24 - Day25-35 |
| ko03020; RNA polymerase | 0.001621551 | 0.011322937 | Day25-35 | Day15-24 - Day25-35 |
| ko04975; Fat digestion and absorption | 0.001226767 | 0.011322937 | Day15-24 | Day15-24 - Day25-35 |
| ko00300; Lysine biosynthesis | 0.002383987 | 0.019852813 | Day15-24 | Day15-24 - Day25-35 |
| ko00643; Styrene degradation | 0.002090647 | 0.019852813 | Day15-24 | Day15-24 - Day25-35 |
| ko00270; Cysteine and methionine metabolism | 0.002443962 | 0.021979557 | Day15-24 | Day15-24 - Day25-35 |
| ko02040; Flagellar assembly | 0.002697924 | 0.021979557 | Day15-24 | Day15-24 - Day25-35 |
| ko05010; Alzheimers disease | 0.002903976 | 0.021979557 | Day15-24 | Day15-24 - Day25-35 |
| ko00280; Valine, leucine and isoleucine degradation | 0.003030708 | 0.030943195 | Day15-24 | Day15-24 - Day25-35 |
| ko00360; Phenylalanine metabolism | 0.003833813 | 0.030943195 | Day15-24 | Day15-24 - Day25-35 |
| ko00473; D-Alanine metabolism | 0.003743341 | 0.030943195 | Day25-35 | Day15-24 - Day25-35 |
| ko00626; Naphthalene degradation | 0.003856739 | 0.040363392 | Day15-24 | Day15-24 - Day25-35 |
